# Effect of *BDNF* Val66Met on hippocampal subfields volumes and compensatory interaction with *APOE*-ε4 in middle-age cognitively unimpaired individuals from the ALFA study

**DOI:** 10.1007/s00429-020-02125-3

**Published:** 2020-08-17

**Authors:** Natalia Vilor-Tejedor, Grégory Operto, Tavia E. Evans, Carles Falcon, Marta Crous-Bou, Carolina Minguillón, Raffaele Cacciaglia, Marta Milà-Alomà, Oriol Grau-Rivera, Marc Suárez-Calvet, Diego Garrido-Martín, Sebastián Morán, Manel Esteller, Hieab H. Adams, José Luis Molinuevo, Roderic Guigó, Juan Domingo Gispert, for the ALFA Study

**Affiliations:** 1 Centre for Genomic Regulation (CRG), The Barcelona Institute for Science and Technology, C. Doctor Aiguader 88, Edif. PRBB, 08003 Barcelona, Spain; 2 Barcelonaβeta Brain Research Center (BBRC), Pasqual Maragall Foundation, Barcelona, Spain; 3 Erasmus MC University Medical Center Rotterdam, Department of Clinical Genetics, Rotterdam, The Netherlands; 4 Universitat Pompeu Fabra (UPF), Barcelona, Spain; 5 CIBER Fragilidad y Envejecimiento Saludable (CIBERFES), Madrid, Spain; 6 IMIM (Hospital del Mar Medical Research Institute), Barcelona, Spain; 7 Centro de Investigación Biomédica en Red de Bioingeniería, Biomateriales y Nanomedicina (CIBER-BBN), Madrid, Spain; 8 Department of Epidemiology, Harvard T.H. Chan School of Public Health, Boston, MA USA; 9 Cancer Epidemiology Research Program, Catalan Institute of Oncology (ICO), Hospitalet de Llobregat, Barcelona, Spain; 10 Servei de Neurologia, Hospital del Mar, Barcelona, Spain; 11 Cancer Epigenetics and Biology Program (PEBC), Bellvitge Biomedical Biomedical Research Institute (IDIBELL), Barcelona, Spain; 12 Josep Carreras Leukaemia Research Institute (IJC), Badalona, Barcelona, Spain; 13 Institució Catalana de Recerca i Estudis Avançats (ICREA), Barcelona, Spain; 14 Physiological Sciences Department, School of Medicine and Health Sciences, University of Barcelona (UB), Barcelona, Spain; 15 Erasmus MC University Medical Center Rotterdam, Department of Epidemiology, Rotterdam, The Netherlands; 16 Erasmus MC University Medical Center Rotterdam, Department of Radiology, Rotterdam, The Netherlands

**Keywords:** *APOE*-ε4, *BDNF*, Hippocampal subfields, Imaging genetics, Subiculum, Val66Met

## Abstract

**Background:**

Current evidence supports the involvement of brain-derived neurotrophic factor (*BDNF*) Val66Met polymorphism, and the ε4 allele of *APOE* gene in hippocampal-dependent functions. Previous studies on the association of Val66Met with whole hippocampal volume included patients of a variety of disorders. However, it remains to be elucidated whether there is an impact of *BDNF* Val66Met polymorphism on the volumes of the hippocampal subfield volumes (HSv) in cognitively unimpaired (CU) individuals, and the interactive effect with the *APOE*-ε4 status.

**Methods:**

*BDNF* Val66Met and *APOE* genotypes were determined in a sample of 430 CU late/middle-aged participants from the ALFA study (ALzheimer and FAmilies). Participants underwent a brain 3D-T1-weighted MRI scan, and volumes of the HSv were determined using Freesurfer (v6.0). The effects of the *BDNF* Val66Met genotype on the HSv were assessed using general linear models corrected by age, gender, education, number of *APOE*-ε4 alleles and total intracranial volume. We also investigated whether the association between *APOE*-ε4 allele and HSv were modified by *BDNF* Val66Met genotypes.

**Results:**

*BDNF* Val66Met carriers showed larger bilateral volumes of the subiculum subfield. In addition, HSv reductions associated with *APOE*-ε4 allele were significantly moderated by *BDNF* Val66Met status. *BDNF* Met carriers who were also *APOE*-ε4 homozygous showed patterns of higher HSv than *BDNF* Val carriers.

**Conclusion:**

To our knowledge, the present study is the first to show that carrying the *BDNF* Val66Met polymorphisms partially compensates the decreased on HSv associated with *APOE*-ε4 in middle-age cognitively unimpaired individuals.

**Electronic supplementary material:**

The online version of this article (10.1007/s00429-020-02125-3) contains supplementary material, which is available to authorized users.

## Introduction

Brain-derived neurotrophic factor (BDNF) is a neurotrophin involved in neurogenesis and synaptic plasticity in the central nervous system, especially in the hippocampus, and has been implicated in the pathophysiology of several neuropsychiatric disorders (Bathina and Das 2015; Autry and Monteggia 2012; Numakawa et al. 2018). The single nucleotide polymorphism (SNP) rs6265 (also known as Val66Met), causes a valine (Val) to methionine (Met) substitution at codon 66 of BDNF protein. Particularly, the study of Val66Met polymorphism within the *BDNF* gene is of special interest because of its documented impact on hippocampal-dependent functions (Notaras and van den Buuse 2018; Toh et al. 2018; Egan et al. 2003; Hariri et al. 2003). Hence, extensive research focuses on the discovery of associations between *BDNF* Val66Met polymorphism and several hippocampal phenotypes. However, recent meta-analyses addressing hippocampal volumes for *BDNF* Val66Met have reported inconsistent statistically significant associations, as well as inconsistencies regarding the direction of the genotype effects across individual studies (Harrisberger et al. 2014, 2015).

Two recent large meta-analyses suggest that the analysis of hippocampal subfield volumes may allow for more accurate detection of genetic effects in genetic association analyses, compared with whole hippocampal volume (van der Meer et al. 2018; Hibar et al. 2017). Moreover, previous studies have shown that different pathological conditions affect subfields differently (West et al. 1994; Jin et al. 2004; Ezzati et al. 2014; Mueller et al. 2010; Hett et al. 2018). In fact, the proven differential expression of BDNF and its receptors in different regions of the hippocampus (Kowiański et al. 2018; Vilar and Mira 2016; Franzmeier et al. 2019), reinforces distinct biological functions of *BDNF* Val66Met polymorphism on the different subfields. However, to our knowledge no previous studies have addressed the effects of the *BDNF* Val66Met polymorphism on hippocampal subfields in cognitively unimpaired (CU) individuals. Most of the studies addressing the association of *BDNF* Val66Met polymorphism and hippocampal volumes (subfields and/or whole hippocampus) included patients of a variety of neuropsychiatric disorders, such as major depressive disorder, schizophrenia and bipolar disorder (Zeni et al. 2016; Cao et al. 2016; Reinhart et al. 2015; Aas et al. 2014; Frodl et al. 2014), showing also inconsistencies concerning the impact of the *BDNF* Val66Met polymorphisms (Tsai 2018).

The ε4 allele of apolipoprotein E *(APOE)* gene, the major genetic risk factor for Alzheimer’s disease (AD) (Mueller and Weiner 2009), has also an impact on hippocampal subfields. *APOE* ε4-carriers have reduced volume of the subicular/CA1 region in AD patients (Pievani et al. 2011), as well as in a pool of older adults that included healthy controls and patients with amnestic mild cognitive impairment (aMCI) and AD dementia, after controlling for the diagnostic group (Kerchner et al. 2014). In a recent report in CU participants, we also showed that *APOE*-ε4 relates to significantly reduced hippocampal tail in a gene dose-dependent manner (Cacciaglia et al. 2018a).

Moreover, recent evidence suggests that *APOE* genotypes differentially affects the expression of *BDNF* through the regulation of its maturation in human astrocytes and its secretion (Sen, Nelson, and Alkon 2015). Astrocytes are known to synthesise BDNF, and as brain APOE is primarily produced by astrocytes, studying APOE and BDNF modulation becomes important. Specifically, interactions between *APOE*-ε4 and *BDNF* have been suggested to influence their secondary effects on AD pathology (Álvarez et al. 2014), and their influence on hippocampal volume (Li et al. 2016; Shi et al. 2014; Liu et al. 2015a, b). In addition, a significant combined effect of *APOE*-ε4 and *BDNF* Val66Met polymorphisms has been reported to moderate β-amyloid-related cognitive decline in preclinical AD (Lim et al. 2015). Episodic memory performance was also found to be impaired in MCI/AD individuals who were also carriers of both the *APOE*-ε4 and *BDNF* Met polymorphisms (Gomar et al. 2016), as well as in healthy individuals (Ward et al. 2014). Overall, evidence suggests biological interactions between *APOE* and *BDNF* for memory and other brain-related processes that may help to explain the increased AD risk in *APOE*-ε4 carriers during the period that precedes the development of symptoms.

Therefore, the aim of the present study is to evaluate the impact of Val66Met polymorphism on hippocampal subfields in a large sample of in middle-age cognitively unimpaired individuals CU participants and to assess whether an interactive effect with the *APOE*-ε4 genotype exists.

## Materials and methods

### Study population and setting

**Fig. 1:**
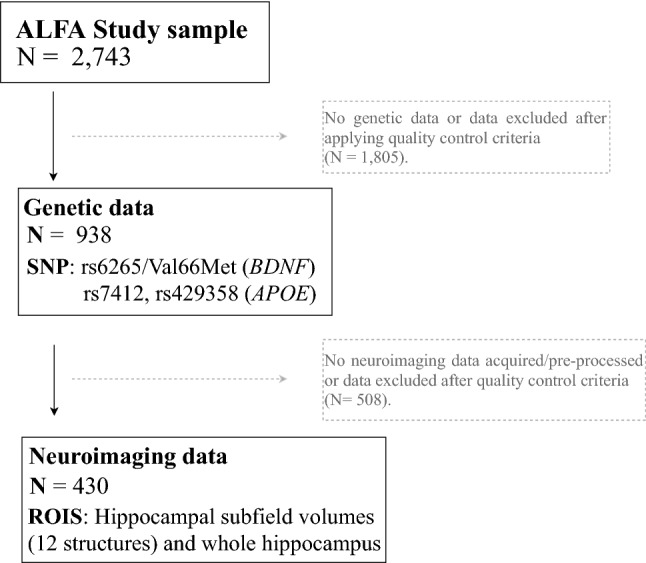
Flow chart depicting the final sample size of the real application. Solid lines and boxes represent individuals remaining in the study. Dashed lines and boxes represent individuals excluded. Reason and number of individuals excluded is indicated in dashed boxes. *SNP* single nucleotide polymorphism, *N* size of the sample, *ROIS* brain regions of interest

Participants were drawn from the ALFA study (Alzheimer and FAmilies) established at the Barcelonaβeta Brain Research Center (Molinuevo et al. 2016), which aims at identifying the neuroimaging and cognitive signatures in preclinical AD. The ALFA study (Clinicaltrials.gov Identifier: NCT01835717) entangles a cohort of 2,743 cognitively unimpaired participants, mostly adult children of patients with AD, and aged between 45 and 75 years. Cognitive status was assessed at baseline as follows: Mini-Mental State Examination (Folstein et al. 1975; Blesa et al. 2001) > 26, Memory Impairment Screen (Buschke et al. 1999; Böhm et al. 2005) > 6, Time-Orientation subtest of the Barcelona Test II (Quinones-Ubeda 2009) > 68, semantic fluency (Ramier and Hecaen 1970; Peña-Casanova et al. 2009) (animals) > 12 and Clinical Dementia Rating scale (Morris 1993) = 0. A subset of 430 participants from the ALFA study with available information on *BDNF* Val66Met polymorphisms and *APOE* genotypes, as well as neuroimaging data (HSv) were included in this study (Fig. 1). The cognitive status of these participants was reviewed if cognitive testing had not been conducted in the last 6 months. For this, mild cognitive impairment (MCI) was ruled out by clinical judgment after interview and accounting for psychometric scores in the main variables of the Free and Cued Selective Reminding Test [FCSRT] (Buschke et al. 2017). The study was conducted in accordance with the directives of the Spanish Law 14/2007, of 3rd of July, on Biomedical Research (Ley 14/2007 de Investigación Biomédica). All participants accepted the study procedures by signing an informed consent form. A subset of 430 participants from the ALFA study with available information on *BDNF* Val66Met polymorphisms and *APOE* genotypes, as well as neuroimaging data (HSv) were included in this study (Fig. 1).

### Genotyping

DNA samples were obtained from whole blood samples by applying salting out protocol. DNA was eluted in 800 µl of H2O (milliQ) and quantified using Quant-iTT PicoGreen® dsDNA Assay Kit (Life Technologies). Integrity of DNA was checked in a subset of samples by running a 1% agarose gel. All the samples were within specification. Genome-wide genotyping was performed using the NeuroChip backbone(Blauwendraat et al. 2017), based on a genome-wide genotyping array (Infinium HumanCore-24 v1.0) containing 306,670 tagging variants and a custom content that has been updated and extended with 179,467 neurodegenerative disease-related variants at the Cancer Epigenetics and Biology Program (PEBC; IDIBELL). Previous step was to normalize the quantity of DNA from each sample. The analysis was performed by the GenomeStudio (Illumina) software using the genotyping module (standard analysis). PLINK was used for genetic data quality control (Purcell et al. 2007). We applied the following sample quality control thresholds: sample call rate > 97% (*N* = 6 exclusion) and heterozygosity 5 SD (*N* = 8 exclusions). Then, we checked sex discordances (*N* = 4 exclusions). In total, we excluded 18 subjects (less than 2%). None of the individuals presented autosomal dominant mutations in *APP*, *PSEN1*, and *PSEN2.* The final genetic data set consisted of volunteers of European ethnic origin with available information regarding *BDNF* Val66Met polymorphism and the *APOE* rs429358 and rs7412 polymorphisms. Genotype and allele frequencies of Val66Met, rs429358 and rs7412 polymorphisms were determined. Moreover, allele frequencies were inspected for potential covariate-related differences. Departures from Hardy–Weinberg equilibrium were also examined (Ryckman and Williams 2008). The *APOE* allelic variants were obtained from allelic combinations of the rs429358 and rs7412 polymorphism (Radmanesh et al. 2014). According to the genotypes of these polymorphisms, subjects were classified depending on the number of ε4 alleles (non-carriers, one ε4 allele or two ε4 alleles).

### Image acquisition and extraction of hippocampal subfield volumes

**Fig. 2:**
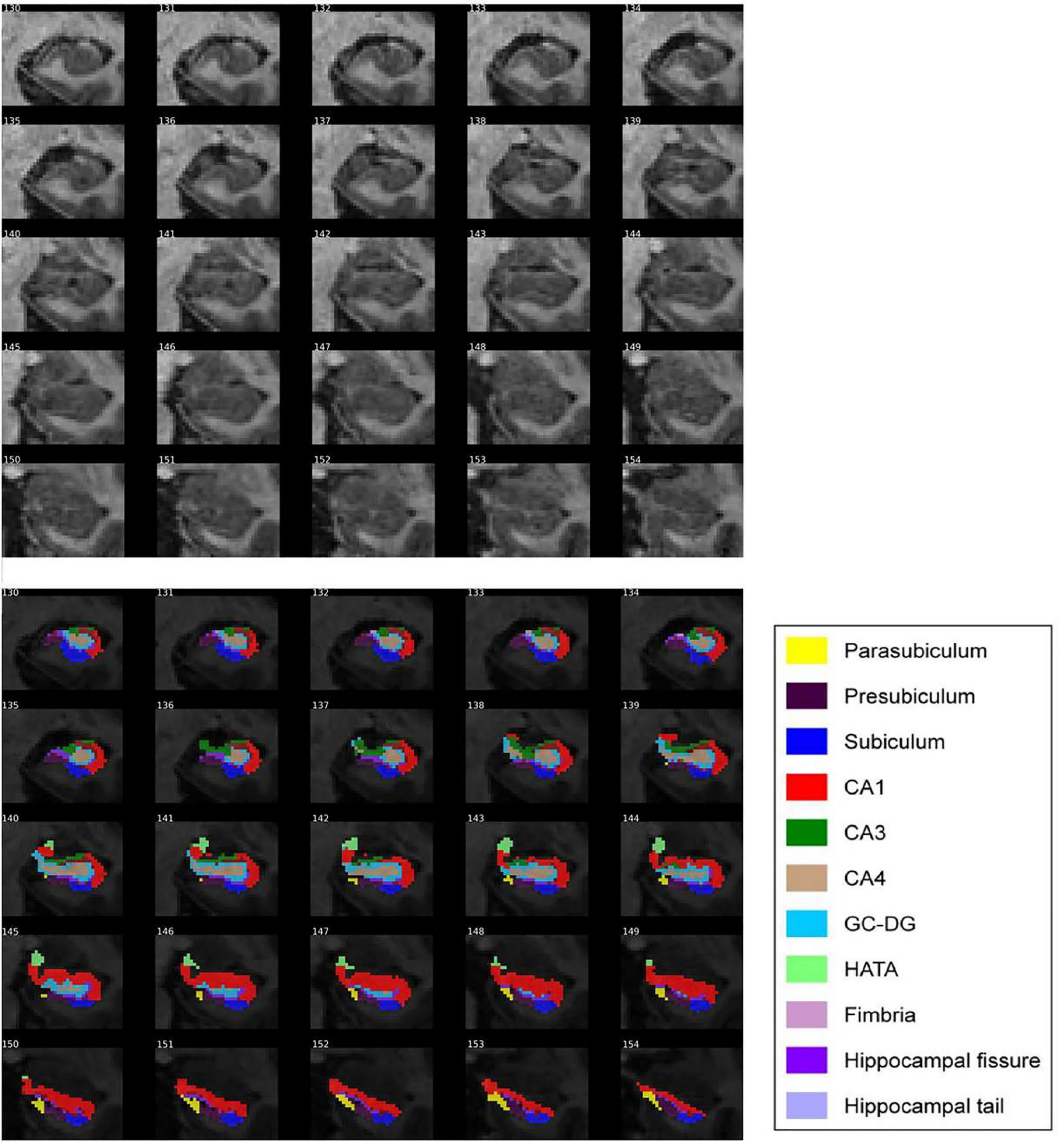
T1 images of hippocampal segmentation

Scans were obtained with a 3 T scanner (Philips Ingenia CX). The MRI protocol was identical for all participants and included high-resolution three-dimensional structural images weighted in T1 with an isotropic voxel of 0.75 × 075 × 0.75 mm^3^. The acquisition parameters were TR/TE/TI = 9.9/4.6/900 ms, flip angle = 8° and a matrix size of 320 × 320 × 240. Hippocampal subfields were segmented using FreeSurfer version 6.0 (Iglesias et al. 2015). We extracted raw volumes for 12 different HSv per hemisphere: the cornu ammonis region 1 (CA1), cornu ammonis region 2/3 (CA3), cornu ammonis region 4 (CA4), dentate gyrus (DG), fimbria, hippocampal-amygdaloid transition area (hata), tail, parasubiculum, presubiculum, subiculum, fissure and molecular layer. The value of the subfields used as the outcomes of the study were calculated as the sum of the regional value of each hemisphere (mm^3^). We visually inspected the segmentation of the individuals included in the study (Fig. 2), and we removed outliers and/or abnormal hippocampal subfields volume values. The whole hippocampal volume, as well as, total intracranial volume were also calculated using Freesurfer (v. 6.0).

### Statistical analysis

Differences in demographic variables were tested using *χ*^2^ test and *F* test for gender, age, education, number of *APOE*-ε4 carriers and total intracranial volume (TIV). The additive, dominant, recessive, and codominant effects of the *BDNF* Val66Met genotype on the hippocampal subfields volume were assessed using general linear models corrected by age, sex, years of education, number of *APOE*-ε4 alleles and TIV. These covariates were selected based on previous associations reported using the ALFA study sample (Cacciaglia et al. 2018b). In brief, the genetic additive model predicts a linear increase of the phenotypic variable depending on the number of Met alleles, whereas the codominant genetic model infers that the heterozygote mean differs from both the homozygote means. The dominant genetic model assumes a common response to 1 or 2 copies of the Met allele. Finally, a recessive genetic model predicts a common response to 0 or 1 copies of the Met allele.

The assumption of different genetic models was performed to counteract a misspecification of the true underlying genetic model, which could have an adverse effect on the statistical power of an association, and on the effect size (Gaye and Davis 2017). The goodness-of-fit of each genetic model was evaluated based on the Akaike information criterion (AIC), for which lower numerical values indicate a better fit of the model (Akaike 1998).

We also investigated whether the association between *BDNF* Val66Met and hippocampal subfield volumes was modified by the number of *APOE*-ε4 alleles, with a second model that included an interaction term between *BDNF* Val66Met polymorphism and the number of ε4 alleles, co-varying for age, sex, years of education and total intracranial volume potential confounders. In this model, dominant genetic effects were assumed for Val66Met polymorphism and additive genetic effects for *APOE*-ε4 alleles.

Moreover, in post-hoc analyses, we evaluated the effects of *BDNF* Val66Met and *APOE*-ε4 status on cognitive performance.

Statistical significance was set at False Discovery Rate (FDR) corrected *p* value < 0.05, and all statistical analyses and data visualization were carried out using R version 3.4.4.

## Results

### Demographic characteristics

Descriptive data of the demographic and *BDNF* Val66Met polymorphism information are presented in Tables 1 and 2. The mean age of the population was 57.1 ± 5.7 years old, with 61.4% women. The *BDNF* Val66Met genotype groups did not significantly differ in the distribution of gender (*χ*^2^[2] = 0.55, *p*: 0.51), number of *APOE*-ε4 alleles (*χ*^2^[2] = 6.13, *p* = 0.19), age (*F*[2,427] = 0.67, *p*: 0.76), years of education (*F*[2,247] = 0.107, *p*: 0.9), or total intracranial volume (TIV) (*F*[2,427] = 1.66, *p*: 0.19). The distribution of *BDNF* Val66Met and *APOE* rs429358 and rs7412 polymorphisms did not deviate from Hardy–Weinberg equilibrium (*χ*^2^[1] = 0.42, *p*: 0.51). Table 3 summarizes the hippocampal subfield volumes analyzed in the study by *BDNF* genotype. All morphometric subfield measures were normally distributed (Kolmogorov Smirnov test, FDR > 0.05) and their variances were homogenous (Levene’s test, FDR > 0.05). Figure S1 shows the pattern of correlation (Pearson correlation statistics) among all subfields included in the study. Hippocampal subfield structures present high correlation among them (*r* > 0.8) (i.e., structural covariance).

**Table 1:** Characteristics of the study according to rs6265 (Val/Met) status

	ValVal carriers (*n *= 247)	ValMet carriers (*n* = 161)	MetMet carriers (*n* = 22)	Total (*n* = 430)	Statistic	*p*
Age (m ± SD; years)	57.31 (5.63)	56.93 (5.84)	55.97 (5.82)	57.1 (5.72)	*F* (2.427) = 0.67	0.76
Sex (female), *n* (%)	154 (62.35%)	98 (60.87%)	12 (54.55%)	264 (61.4%)	Chi (2) = 0.549	0.512
Education (m ± SD; years)	13.87 (± 3.53)	14.03 (± 3.53)	13.95 (± 3.5)	13.93 (± 3.52)	*F* (2.427) = 0.107	0.899
Number of *APOE*-ε4 alleles, *n* (%)	0: 160 (64.78%); 1: 75 (30.36%); 2: 12 (4.86%)	0: 89 (55.28%); 1: 59 (36.65%); 2: 13 (8.07%)	0: 12 (54.55%); 1: 7 (31.82%); 2: 3 (13.64%)	0: 261 (60.7%); 1: 141 (32.79%); 2: 28 (6.51%)	Chi (2) = 6.129	0.19
TIV (m ± SD; cm^3^)	1442.91 (± 177.13)	1453.1 (± 163.66)	1511.62 (± 163.28)	1450.24 (± 171.79)	*F* (2.427) = 1.656	0.192

Mean and SD are shown for continuous variables

*n* sample size, *m* mean, *SD* standard deviation, *TIV* total intracranial volume *P* p value

**Table 2:** Characteristics of *BDNF* Val66Met and *APOE* polymorphisms

Gene	SNP	CHR	position	Allele 1	Allele 2	MAF	MAF gp*	Genotype distribution	HWE	Array**
*BDNF*	Val66Met (rs6265)	11	27,658,369 (CRCh37)	C [Val]	T [Met]	0.238	0.19,437	ValVal: 247 / ValMet: 161 / MetMet: 22	0.51	Neurochip backbone (Infinium HumanCore-24 v1.0)
								Allele ε4 distribution		
*APOE*	rs429358	19	45,411,941 (CRCh37)	T	C	0.214	0.138	ε4-non carriers: 261; ε4 heterozygous: 141; ε4 homozygous: 28	0.137	
	rs7412	19	45,412,079 (CRCh37)	C	T	0.043	0.061			

*SNP* single nucleotide polymorphisms, *BP* base position, *A1* major allele, *A2* minor allele, *MAF*  minor allele frequency, MAF gp*  MAF general population. Source: *gnomAD* genome aggregation database, *HWE* Hardy weinberg equilibrium

Array source** Blauwendraat et al., NeuroChip, an updated version of the NeuroX genotyping platform to rapidly screen for variants associated with neurological diseases. Neurobiology of Aging. 2017 vol: 57 pp: 247.e9-247.e13

**Table 3:** Characteristics of hippocampal subfield volumes

Hippocampal subfield	ValVal carriers (*n* = 247)				ValMet carriers (*n* = 161)				MetMet carriers (*n* = 22)				Total (*n* = 430)			
	Mean (SD)	Min	Median	Max	Mean (SD)	Min	Median	Max	Mean (SD)	Min	Median	Max	Mean (SD)	Min	Median	Max
CA1, mm^3^	1193 (123)	920	1176	1595	1214 (119)	950	1207	1518	1255 (161)	924	1250	1630	1204 (124)	920	1192	1630
CA3, mm^3^	361 (42)	241	357	511	364 (40)	273	361	475	385 (62)	299	375	525	364 (43)	241	358	525
CA4, mm^3^	454 (44)	299	448	594	459 (38)	374	458	564	480 (59)	377	464	619	457 (43)	299	455	619
GC-ML-DG, mm^3^	535 (52)	351	530	706	541 (45)	432	535	656	566 (67)	450	553	724	539 (51)	351	533	724
Subiculum, mm^3^	802 (91)	594	804	1113	822 (86)	639	816	1067	866 (104)	688	859	1061	812 (91)	594	809	1113
Presubiculum, mm^3^	601 (69)	412	599	797	614 (60)	482	616	786	630 (75)	528	616	781	608 (66)	412	604	797
Parasubiculum, mm^3^	125 (18)	83	123	194	127 (17)	92	125	175	131 (21)	94	127	194	126 (18)	83	125	194
Hippocampal fissure, mm^3^	339 (45)	214	338	514	345 (40)	255	346	448	360 (50)	293	348	457	343 (44)	214	341	514
Hippocampal tail, mm^3^	1068 (126)	767	1058	1388	1086 (128)	808	1087	1488	1080 (133)	787	1069	1328	1076 (127)	767	1066	1488
Fimbria, mm^3^	174 (30)	108	173	308	175 (30)	105	175	267	182 (29)	132	181	241	175 (30)	105	174	308
Hata, mm^3^	117 (14)	72	116	179	118 (13)	83	117	156	120 (16)	85	119	147	118 (14)	72	117	179
Molecular layer, mm^3^	1059 (99)	767	1052	1311	1077 (93)	889	1070	1328	1121 (122)	910	1110	1404	1069 (99)	767	1061	1404
whole hippocampus, mm^3^	6489 (597)	4623	6435	8235	6598 (552)	5407	6567	8014	6817 (747)	5351	6793	8582	6547 (594)	4623	6492	8582

Means, standard deviations (SD), Median, and ranges values are shown

Segmentation of hippocampal subfields performed with FreeSurfer version 6.0 image analysis suite

*CA1* cornu ammonis region 1, *CA3* cornu ammonis region 23, *CA4* cornu ammonis region 4, *GC-ML-DG* granule cells in the molecular layer of the dentate gyrus, *hata* hippocampal-amygdaloid transition region, *HP* hippocampus, *CI95* confidence interval, *FDR95* false discovery rate corrected *p* value < 0.05

### Effect of BDNF Val66Met polymorphism on hippocampal subfields

**Fig. 3:**
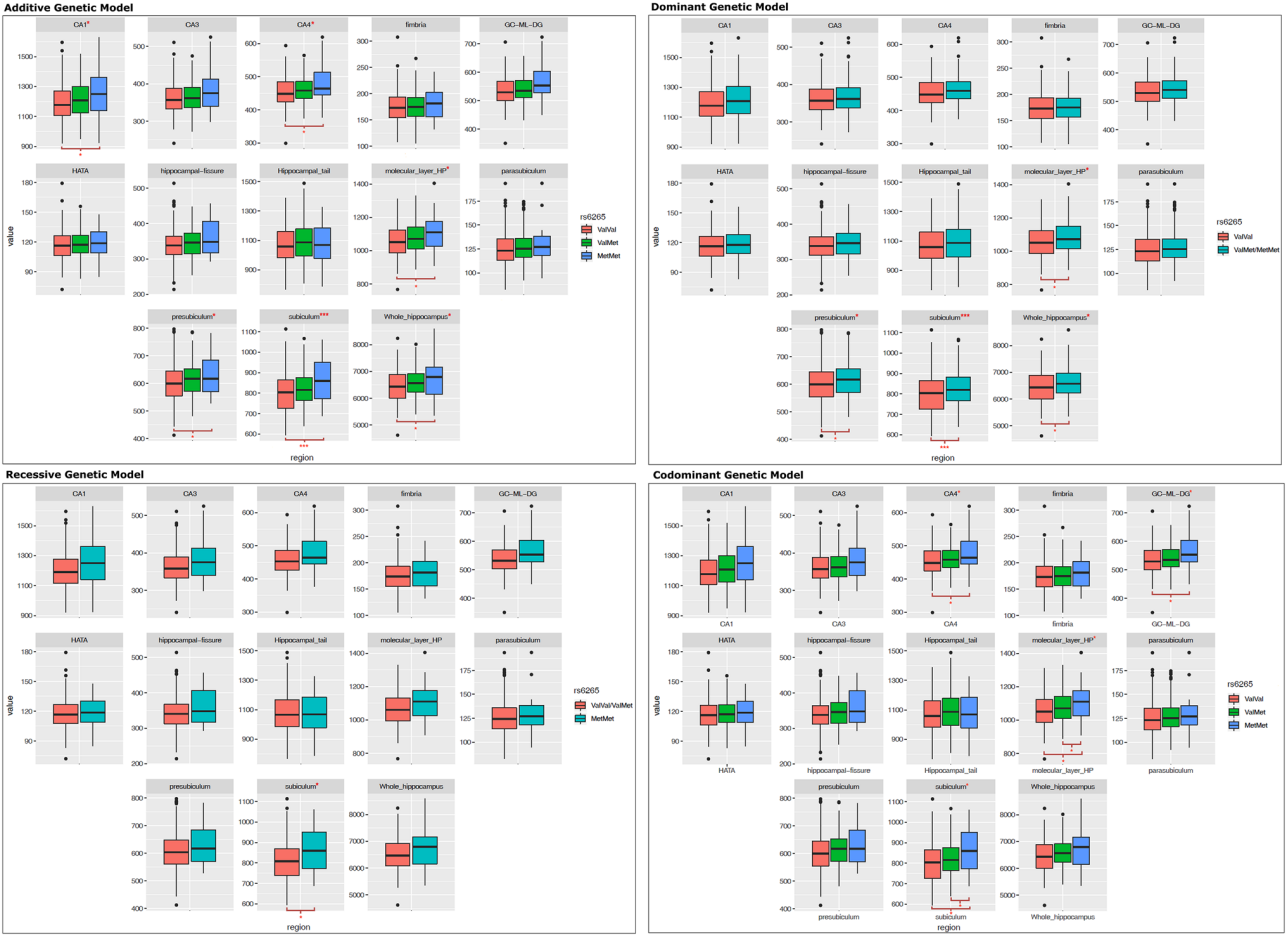
Box plot of change in hippocampal subfield volumes between *BDNF* Val66Met (rs6265) genotypes under additive, dominant, recessive and codominant genetic models. Middle line in box represents the median; lower box bounds the first quartile; upper box bounds the 3rd quartile. Whiskers represent the 95% confidence interval of the mean. Open circles are outliers from 95% confidence interval. *Significant difference between groups at a nominal level (*p* < 0.05). ***Significant difference between groups after multiple comparison correction (*FDR* < 0.05). *CA1* cornu ammonis region 1, *CA3* cornu ammonis region 3, *CA4* cornu ammonis region 4, *GC-ML-DG* granule cells in the molecular layer of the dentate gyrus, *hata* hippocampal-amygdaloid transition region, *HP* hippocampus

General linear models revealed that Met carriers showed statistically significant larger bilateral volumes of the subiculum under the dominant model (${\beta }_{dom}$= 2.53%, ${{p}_{FDR}}_{dom}$ = 3 × ${10}^{-3}$) (Table 4 and Fig. 3). For subiculum subfield, an additive genetic model obtained the lowest AICs score (AIC = 4890.43), indicating that this model is the most parsimonious model for this subfield structure. Moreover, we found statistically significant larger bilateral volumes of the subiculum under the additive genetic model (${\beta }_{add}$= 2.39%, ${p}_{FD{R}_{add}}$= 0.013) (Table S1). No significant results after FDR-correction were found under recessive and codominant genetic models. In addition, nominal significant results without FDR adjustment (*p* < 0.05) showed larger bilateral volumes of the molecular layer of the hippocampus (β: 1.55%, *p*: 0.007), presubiculum (β: 1.74%, *p*: 0.041), and whole hippocampal volume (β: 1.46%, *p*: 0.025) for Met carriers under the dominant genetic model. Results of all adjusted genetic models for each HSv can be found in Table S1.

**Table 4:** Main effects of Val66Met genotype on hippocampal subfields (mm^3^)

Hippocampal subfield	Best genetic model	Effect (mm^3^)	CI 95%	Effect (%)	*p* value	FDR95%	AIC
*CA1*	*Dominant*	18.575	(− 0.67, 37.82)	1.54%	0.059	0.531	5188.564
*CA3*	*Recessive*	14.291	(− 1.43, 30.01)	3.93%	0.076	0.684	4321.049
*CA4*	*Recessive*	14.04	(− 0.69, 28.77)	3.07%	0.062	0.671	4264.9
*GC-ML-DG*	*Recessive*	16.756	(− 0.19, 33.71)	3.11%	0.053	0.636	4385.858
*Subiculum*	*Additive*	19.435	(8.06, 30.81)	2.39%	**0.001**	0.013*	4890.434
*Presubiculum*	*Dominant*	10.556	(0.44, 20.67)	1.74%	**0.041**	0.41	4635.326
*Parasubiculum*	*Dominant*	1.845	(− 1.06, 4.75)	1.46%	0.214	1	3562.288
*Hippocampal fissure*	*Dominant*	5.226	(− 2.05, 12.5)	1.52%	0.16	1	4351.502
*Hippoccampal tail*	*Dominant*	12.37	(− 9.43, 34.17)	1.15%	0.267	1	5295.52
*Fimbria*	*Recessive*	1.601	(− 10.09, 13.29)	0.91%	0.789	1	4065.996
*Hata*	*Dominant*	0.333	(− 1.95, 2.61)	0.28%	0.775	1	3354.183
*Molecular layer*	*Additive*	16.578	(4.59, 28.57)	1.55%	**0.007**	0.084	4937.902
*Whole hippocampus*	*Dominant*	95.904	(12.06, 179.75)	1.46%	**0.025**	0.275	6454.188

All models were adjusted by sex, years of education, number of APOE-ε4 allele and total intracranial volume

*CA1* cornu ammonis region 1, *CA3* cornu ammonis region 23, *CA4* cornu ammonis region 4, *GC-ML-DG* granule cells in the molecular layer of the dentate gyrus, *hata* hippocampal-amygdaloid transition region, *HP* hippocampus, *CI95* confidence interval, *FDR95* false discovery rate corrected *p* value < 0.05. *AIC* akaike information criterion

### Effect of the interaction between APOE-ε4 and BDNF Val66Met on hippocampal subfields

**Fig. 4:**
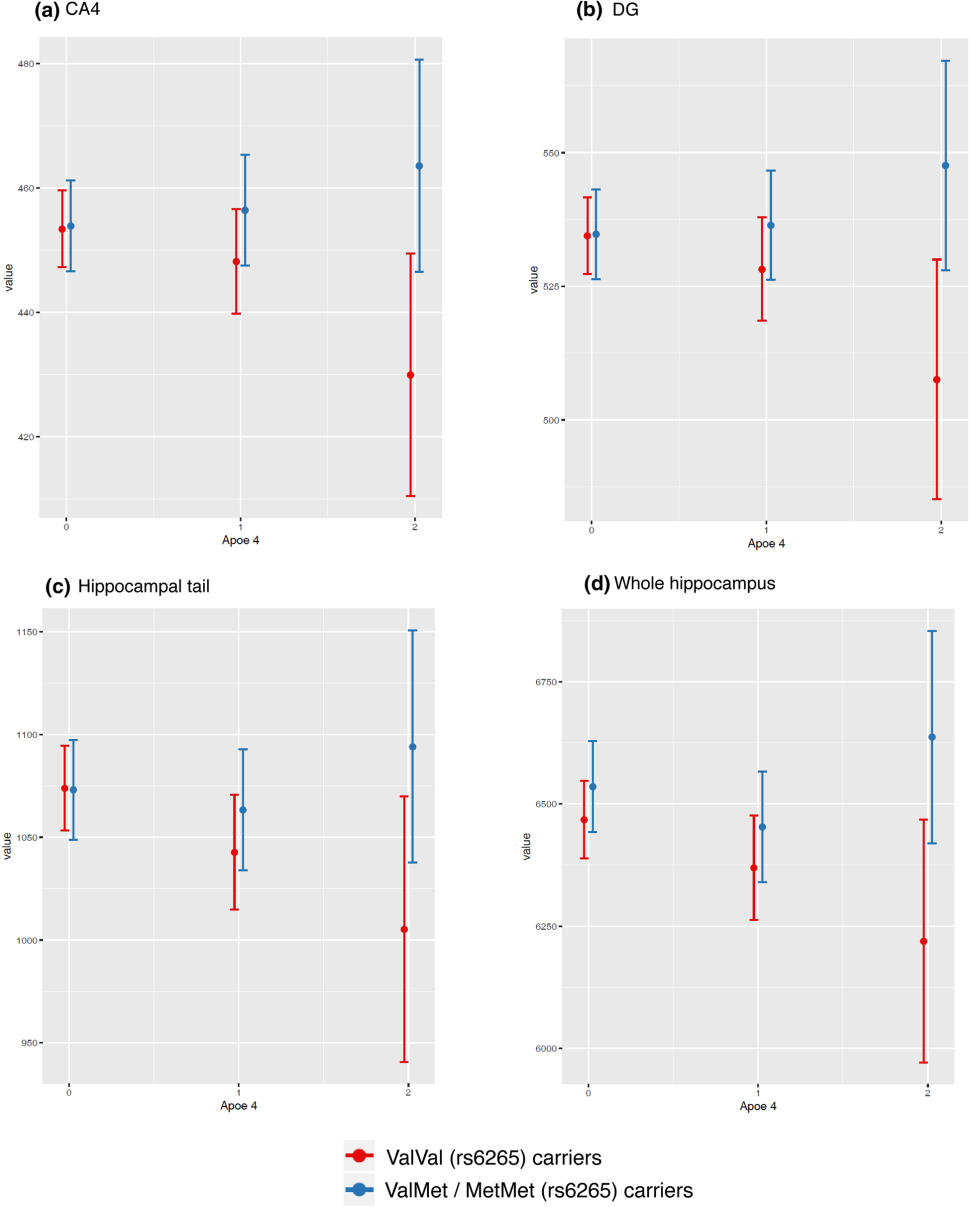
Differences according *BDNF* Val66Met genotypes in associations between *APOE*-ε4 and **a** Cornu ammonis region 4 (CA4) subfield volume, **b** Granule cells in the molecular layer of the dentate gyrus (GC-ML-DG) subfield volume, **c** hippocampal tail subfield volume, and **d** whole hippocampal volume

As expected, *APOE* ε4 allele was associated with lower bilateral volumes of the hippocampal subfields, even though on a trend-level (Table S2). Interestingly, when this association was studied according to *BDNF* Val66Met genotypes, we observed a significant interaction with the presence of Met alleles (*p* < 0.05) (Table 5). *APOE*-ε4 homozygotes carrying at least one Met allele presented nominally significant larger bilateral volumes of the CA4 (β: 7.25%, *p*: 0.016) and DG (β: 7.38%, *p*: 0.012) subfields, hippocampal tail (β: 8.33%, *p*: 0.05), and whole hippocampal volumes (β: 5.35%, *p*: 0.046) than the expected combined effect of the individual contribution of *APOE*-ε4 (reverse effect) and *BDNF* Val66Met (Fig. 4). Moreover, even though the results for the remainder hippocampal subfields were statistically not significant, changes on hippocampal subfields volumes follow the same general patterns (Figure S2).

**Table 5:** Interaction Effects Between number of *APOE*-ε4 alleles (under additive model) and *BDNF* Val66Met polymorphism (under dominant genetic model) on hippocampal subfields

Hippocampal subfield	Dominant genetic model				
	Effect (mm^3^)	CI 95%	Effect (%)	*p *value	FDR95%
*CA1*	− 10.788	(− 52.36, 30.79)	− 0.90%	0.611	1
	55.535	(− 23.33, 134.4)	4.61%	0.168	1
*CA3*	10.889	(− 4.31, 26.09)	2.99%	0.161	1
	27.425	(− 1.4, 56.25)	7.53%	0.063	0.567
*CA4*	7.797	(− 6.39, 21.98)	1.71%	0.282	1
	33.155	(6.25, 60.06)	7.25%	**0.016**	0.192
*GC-ML-DG*	7.94	(− 8.39, 24.27)	1.47%	0.341	1
	39.772	(8.79, 70.75)	7.38%	**0.012**	0.156
*Subiculum*	− 13.434	(− 42.9, 16.03)	− 1.65%	0.372	1
	25.529	(− 30.36, 81.42)	3.14%	0.371	1
*Presubiculum*	0.013	(− 21.92, 21.94)	~ 0%	0.999	1
	13.726	(− 27.87, 55.33)	2.26%	0.518	1
*Parasubiculum*	2.587	(− 3.7, 8.88)	2.05%	0.421	1
	2.78	(− 9.15, 14.71)	2.21%	0.648	1
*Hippocampal fissure*	− 1.375	(− 17.1, 14.35)	− 0.40%	0.864	1
	7.756	(− 22.08, 37.59)	2.26%	0.611	1
*Hippoccampal tail*	21.561	(− 25.48, 68.6)	2.00%	0.369	1
	89.676	(0.45, 178.9)	8.33%	**0.05**	0.506
*Fimbria*	− 7.8	(− 19.07, 3.47)	− 4.46%	0.176	1
	8.845	(− 12.54, 30.23)	5.05%	0.418	1
*Hata*	− 0.304	(− 5.25, 4.64)	− 0.26%	0.904	1
	4.091	(− 5.29, 13.47)	3.47%	0.393	1
*Molecular layer*	− 2.31	(− 33.37, 28.75)	− 0.22%	0.884	1
	49.911	(− 9.01, 108.83)	4.67%	0.098	0.784
*Whole hippocampus*	16.151	(− 164.67, 196.97)	0.25%	0.861	1
	350.445	(7.43, 693.46)	5.35%	**0.046**	0.506

All models were adjusted by sex, years of education, age and total intracranial volume

*CA1* cornu ammonis region 1, *CA3* cornu ammonis region 23 *CA4* cornu ammonis region 4, *GC-ML-DG* granule cells in the molecular layer of the dentate gyrus, *hata* hippocampal-amygdaloid transition region, *HP* hippocampus, *CI95* confidence interval, *FDR95* FDR corrected *p* value < 0.05

### Post-hoc analyses: Effect of *BDNF* Val66Met and *APOE*-ε4 on cognitive performance

The post hoc analyses, although not significant, suggested better cognitive performance patterns for Met carriers in most FCSRT domains, and in depression scores of the Hospital Anxiety and Depression Scale (HADS) (Table 6). In addition, *APOE*-ε4 status did not significantly influence the effects of *BDNF* Val66Met genotypes on cognitive performance.

**Table 6:** Main and interaction effects of *BDNF*-Val66Met genotype and *APOE*-ε4 status (dominant model) on cognitive performance (HADS, FCSRT tests)

Cognition (Test)	Effect	CI 95%	*p* value	FDR-95%
HADS-anxiety				
* Val66Met*	− 0.040	(− 0.29, 0.21)	0.756	
* APOE-ε4*	− 0.188	(− 0.59, 0.22)	0.364	
* Val66Met* x *APOE-ε4*			0.243	1
HADS-depression			0.318	
* Val66Met*	0.036	(− 0.43, 0.5)	0.879	
* APOE-ε4*	0.32	(− 0.36, 0.99)	0.354	
* Val66Met* x *APOE-ε4*			0.318	1
Delayed recall (DR)				
* Val66Met*	0.143	(− 0.29, 0.57)	0,517	
* APOE-ε4*	0.067	(− 0.61, 0.74)	0.847	
* Val66Met* x *APOE-ε4*			0.85	1
Total recall (TR)				
* Val66Met*	0.111	(− 1.22, 1.44)	0.87	
* APOE-ε4*	1.398	(− 0.67, 3.47)	0.188	
* Val66Met* x *APOE-ε4*			0.45	1
Delayed free recall (DFR)				
* Val66Met*	− 0.513	(− 1.30, 0.27)	0.204	
* APOE-ε4*	0.528	(− 0.69, 1.75)	0.4	
* Val66Met* x *APOE-ε4*			0.722	1
Free recall (FR)				
* Val66Met*	2.099	(− 2.4, 6.59)	0.387	
* APOE-ε4*	− 0.853	(− 6.367, 4.662)	0.77	
* Val66Met* x *APOE-ε4*			0.385	1
Retention index				
* Val66Met*	− 0.029	(− 0.091, 0.033)	0.391	
* APOE-ε4*	− 0.008	(− 0.085, 0.068)	0.837	
* Val66Met* x *APOE-ε4*			0.948	1

All models were adjusted by sex, years of education, and total intracranial volume

*CI95* confidence interval, *FDR95* FDR corrected *p* value < 0.05, *HADS* hospital anxiety and depression scale (HADS), *HADS-Anxiety* anxiety score of the HADS, *HADS-Depression* depression score of the HADS, *FCSRT* free and cued selective reminding test

## Discussion

To the best of our knowledge, this is the first study to show a phenotypic effect of the *BDNF* Val66Met polymorphism in the hippocampal subfields of cognitively unimpaired (CU) individuals. Moreover, the present study is also the first in CU to find an effect modification by *BDNF* Val66Met polymorphism of associations between *APOE*-ε4 status and hippocampal subfield volumes.

We first found significantly larger bilateral subiculum volumes in CU middle-aged/late-middle-aged *BDNF* Val66Met carriers in a dose-dependent manner. The direction of the effects is consistent across different subfields and the entire hippocampal formation, as shown by the nominally significant difference between *BDNF* Val66Met carriers and non-carriers involving the molecular layer of the hippocampus, as well as the pre-subiculum and the whole hippocampus.

Given that *BDNF* Val66Met polymorphism has been related to impaired hippocampal long-term potentiation which underlies learning and memory (Spriggs et al. 2018), our results may underline compensatory mechanisms in the Met-carriers to achieve normative episodic recall, which is highly specialized in the subiculum (Eldridge et al. 2005; Suthana et al. 2015). However, although most studies showed that large hippocampal volumes lead to better memory performance and may protect from dementia (Pohlack et al. 2014; Whitwell 2010; Erten-Lyons et al. 2009), the impact of hippocampal volume on cognitive performance in middle-aged CU individual’s remains controversial. For instance, smaller hippocampal volumes have been related to better episodic memory, due to efficient synaptic pruning (Van Petten 2004). Thus, our results could suggest a moderating role of *BDNF* in the neurobiology of hippocampal subfields, which may stress the importance to consider the hippocampal formation at the subfield level to disentangle potential opposite effects leading to the aforementioned conflicting results. In addition, we cannot rule out that the *BDNF* Val66Met polymorphism may differentially influence the morphology of other brain areas. This calls for additional whole-brain voxel-wise studies addressing distinct genetic models of penetrance of *BDNF* Val66Met.

Second, we also observed that Met-carriers compensate for the deleterious impact of the number of *APOE*-ε4 alleles on hippocampal subfield volumes. As expected, we observed that *APOE*-ε4 homozygotes showed a tendency towards displaying reduced volumes of the subiculum and hippocampal tails, in accordance with previous reports (Kerchner et al. 2014; Cacciaglia et al. 2018a; Pievani et al. 2011). These individuals are at increased higher risk (× 15) to develop AD as compared to *APOE*-ε4 non-carriers. The lower hippocampal volumes in *APOE*-ε4 carriers are often interpreted as brain marker that confers vulnerability towards developing the clinical picture of AD. Strikingly, we found that *APOE*-ε4 homozygotes who were also Met-carriers countered the effect of the *APOE* genotype and presented HSv within the ranges expected for *APOE*-ε4 non-carriers, particularly in the CA4, GC-ML-DG and the hippocampal tails. It could be argued that Met-carriers can counter the deleterious effect of the *APOE-*ε4 genotype in the age range of the studied sample.

Another possible explanation to this finding could raise from an interaction of the *BDNF* Val66Met polymorphism with pathological markers of AD, as the *APOE*-ε4 allele has also been strongly linked to a dose-dependent increase in the prevalence of abnormally elevated cerebral amyloid deposition in CU individuals (Reiman et al. 2009). By the mean age of our *APOE*-ε4 homozygote group (56.62 ± 5.71y), about half of them are expected to display abnormally high amyloid levels (Jansen et al. 2015). However, previous longitudinal reports on amyloid-positive CU individuals have described that the *BDNF* Val66Met allele was associated with a steeper decline in cognitive function and hippocampal atrophy (Yen Ying Lim et al. 2013). Moreover, this deleterious effect is more severe in *APOE*-ε4 carriers (Lim et al. 2015). These studies, however, were performed in significantly older cohorts (average age of 70y) than that in our work. Another recent study performed in subjects with an age range similar to ours (55y) confirmed this longitudinal pattern of decline in cognition, particularly in amyloid-positive CU individuals (Boots et al. 2017). However, in this work, Boots et al*.* also reported that, at baseline, Met carriers showed a significantly better cognitive performance (verbal learning and memory, speed and flexibility, and working memory), even if amyloid positive. Similar patterns of effect were observed in our sample. Specifically, we found that Met carriers suggested patterns of better cognitive performance on the Free and Cued Selective Reminding Test (FCSRT) domains, and on the Depression score of the Hospital Anxiety and Depression Scale (HADS). Although these differences were not statistically significant, one potential explanation to reconcile our findings with the existing literature would be that the Met genotype might provide a limited beneficial effect during middle age. When no longer capable of compensating for the deleterious downstream effects of amyloid accumulation, then Met carriers would experience faster hippocampal atrophy and a steeper decline in cognition. Neither shall it be excluded that *APOE*-ε4 and Met carriers could be the most vulnerable to an inflammatory response at the beginning of the amyloid-pathology. Nevertheless, the current unavailability of core AD biomarkers and the cross-sectional nature of this study constitute a limitation for the full interpretation of the interaction between the *BDNF* Met and *APOE*-ε4 genotypes. Nevertheless, this will be mitigated in the longitudinal follow-up of the cohort here studied, as a subset of our participants will undergo a lumbar puncture to assess cerebrospinal fluid levels of core AD biomarkers (Aβ42, total Tau, and phosphorylated Tau).

A strong feature of our study that may sustain our ability to detect a significant effect of Val66Met genotype (and its interaction with *APOE*) on hippocampal subfields is that the studied cohort presents a higher prevalence of *BDNF* Met and *APOE-*ε4 homozygotes compared with previous studies. While most of the studies reported allele frequencies between 0% (studies without MetMet carriers) to 6% (Harrisberger et al. 2015), the minor allele frequency in our study achieves 23%, which is even higher to the population frequency (15–19%, Source: Genome Aggregation Database https://gnomad.broadinstitute.org/variant/11-27679916-C-T). Similarly, the high number of *APOE*-ε4 carriers in the ALFA participants compared to the general population (19% vs. 14%, respectively; *p* < 0.001) has allowed us to disentangle specific effects in ε4 homozygotes, while most studies pool them with heterozygotes in a single *APOE*-ε4 carrier group. In our study, the high prevalence of these less frequent genotypes has allowed us to achieve a relatively higher inferential power, allowing for testing additive, recessive and codominant genetic effects, as well as, gene–gene interactions.

Another substantial strength is the use of a high-resolution T1 scan as compared to previous studies, combined with the use of the most recent version (v.6.0) of the hippocampal subfield segmentation toolbox in Freesurfer, which overcomes significant shortcomings of previous versions (Iglesias et al. 2015; Wisse et al. 2014; Mueller et al. 2018). Thus, subfield volumes available for this analysis are of substantially better quality, which, combined with a considerably higher sample size, has allowed us to achieve a significantly superior statistical power than previous reports. Moreover, only a few studies have assessed hippocampal subfield volumes and compared them to whole hippocampal volumetry, even though the independent genetic variation specific to hippocampal subfields (Elman et al. 2019). Thus, our analyses based on hippocampal subfields increased the sensitivity of the results, which make our study more robust and consistent than previous ones.

Finally, in contrast to previous studies in which the diagnostic value of hippocampal subfield volumes related to Val66Met polymorphism was only assessed by comparing patients with psychiatric disorders, our study includes CU middle-aged/late-middle-age participants. This is also a relevant strength because the misrepresentation of the general population could constitute a bias in the assessment of the diagnostic utility of hippocampal subfield volumes, due to potential etiologies associated with neurodegenerative processes (de Flores et al. 2015).

Altogether, our findings suggest that the *BDNF* Met allele might confer a time-limited resilience, which protects the hippocampi from the downstream deleterious effects of ageing and/or amyloid accumulation, thus mediating the risk effect of *APOE*-ε4. Hence, these results prompt us to further explore hippocampal atrophy rates and cognitive trajectories of *BDNF* Met carriers compared to Val homozygotes, also as a function of their *APOE* genotype and long-term accumulation of amyloid beta.

## Electronic supplementary material

Below is the link to the electronic supplementary material.

Supplementary file1 (PDF 13 kb)Figure S1. Heatmap showing pair correlations across brain structures of ALFA project subsample, with blue color indicating positive correlations and red colour indicating negative correlations. Legend: CA1, cornu ammonis region 1; CA23, cornu ammonis region 23; CA4, cornu ammonis region 4; GC-ML-DG, granule cells in the molecular layer of the dentate gyrus; hata, hippocampal-amygdaloid transition region; HP, hippocampus.

Supplementary file2 (PDF 257 kb) Figure S2. Differences according BDNF Val66Met genotypes in associations between APOE-ε4 and the remaining subfields. Legend: CA1, cornu ammonis region 1; CA23, cornu ammonis region 23; hata, hippocampal-amygdaloid transition region.

Supplementary file3 (DOCX 21 kb) Table S1. Main effects of Val66Met genotype on hippocampal subfields (mm3). All models were adjusted by sex, years of education, number of APOE-e4 allele and total intracranial volume.

Supplementary file4 (DOCX 14 kb) Table S2. Main effects of number of APOE-e4 alleles on hippocampal subfields (mm3). All models were adjusted by sex, years of education, age, val66Met genotypes and total intracranial volume.
