# Supplementary figures and images for: Effect of BDNF Val66Met on hippocampal subfields volumes and compensatory interaction with APOE-ε4 in middle-age cognitively unimpaired individuals from the ALFA study
Source: Brain Struct Funct. 2020 Aug 17;225(8):2331–45. doi: 10.1007/s00429-020-02125-3 (PMC7544723; doi:10.1007/s00429-020-02125-3)

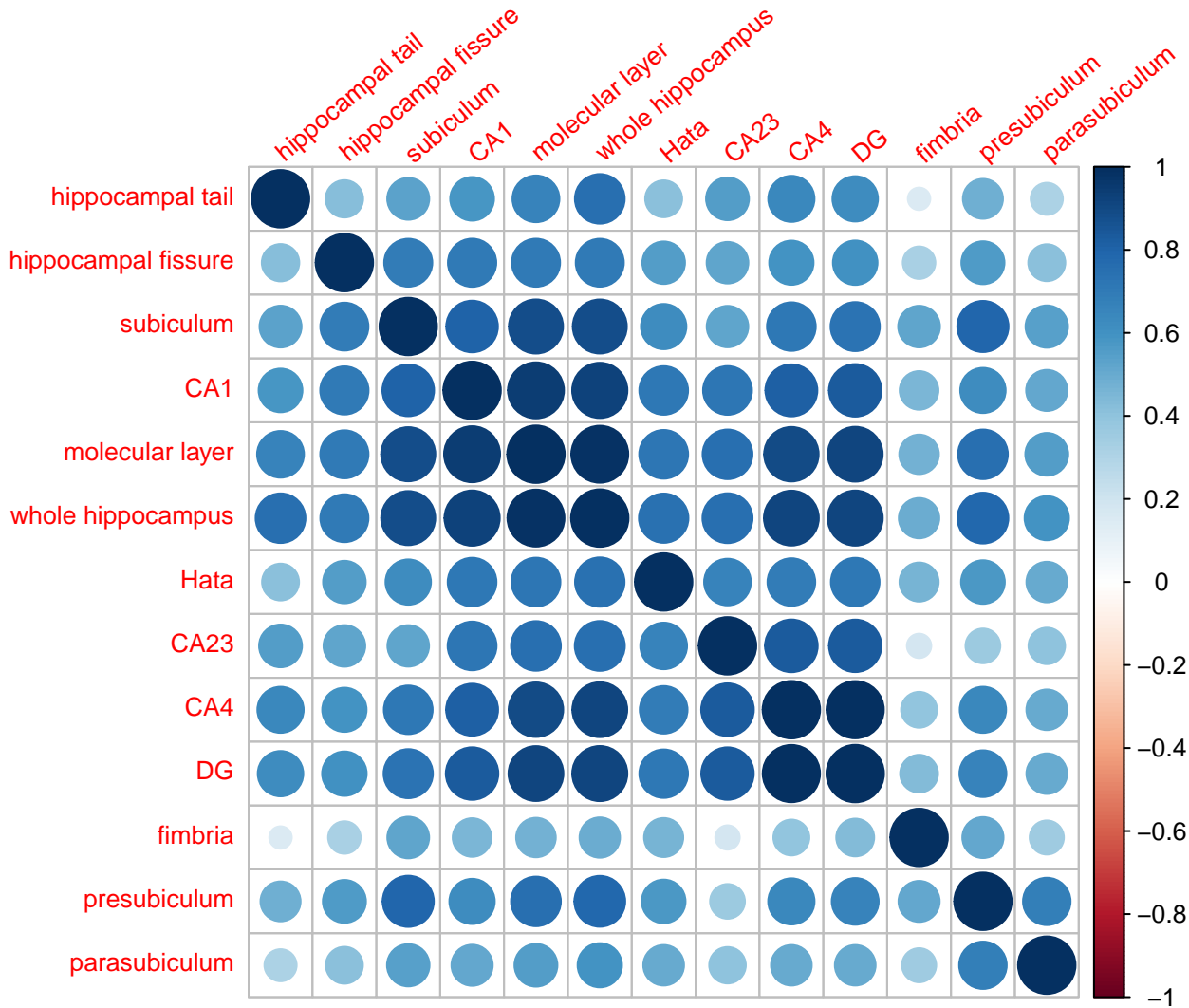

Supplement: Supplementary file 1 — Supplementary file1 (PDF 13 kb)Figure S1. Heatmap showing pair correlations across brain structures of ALFA project subsample, with blue color indicating positive correlations and red colour indicating negative correlations. Legend: CA1, cornu ammonis region 1; CA23, cornu ammonis region 23; CA4, cornu ammonis region 4; GC-ML-DG, granule cells in the molecular layer of the dentate gyrus; hata, hippocampal-amygdaloid transition region; HP, hippocampus. [file 429_2020_2125_MOESM1_ESM.pdf]

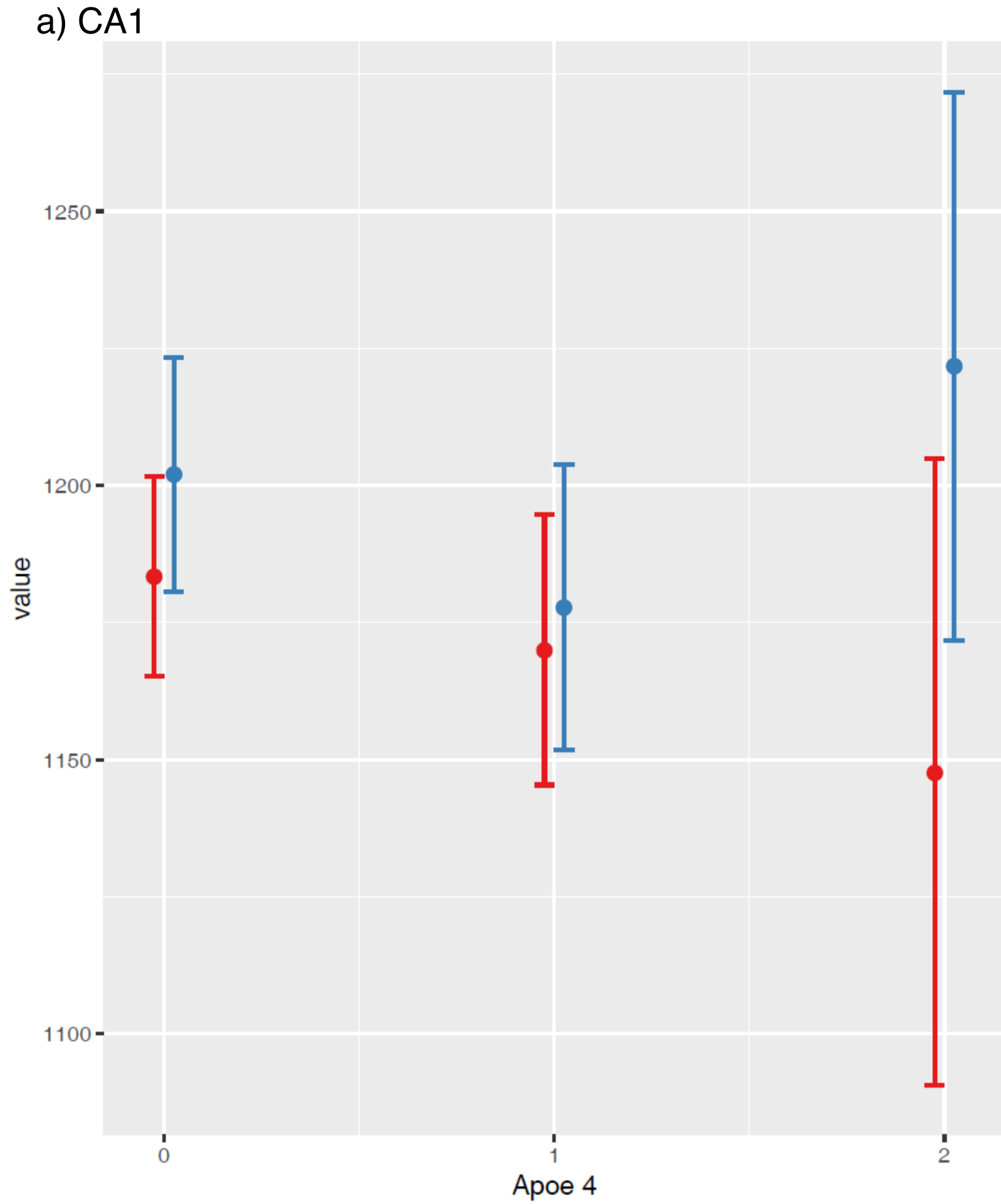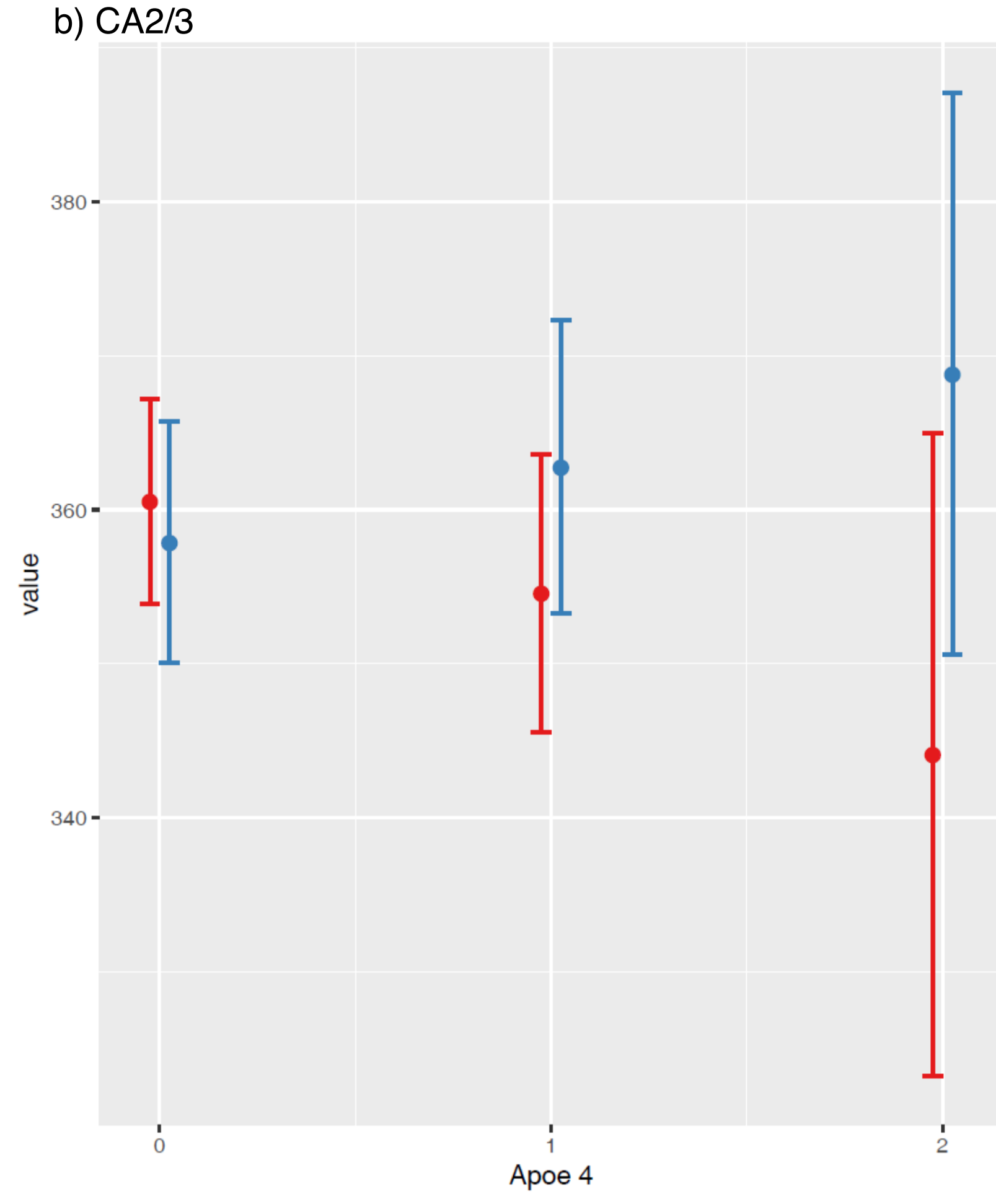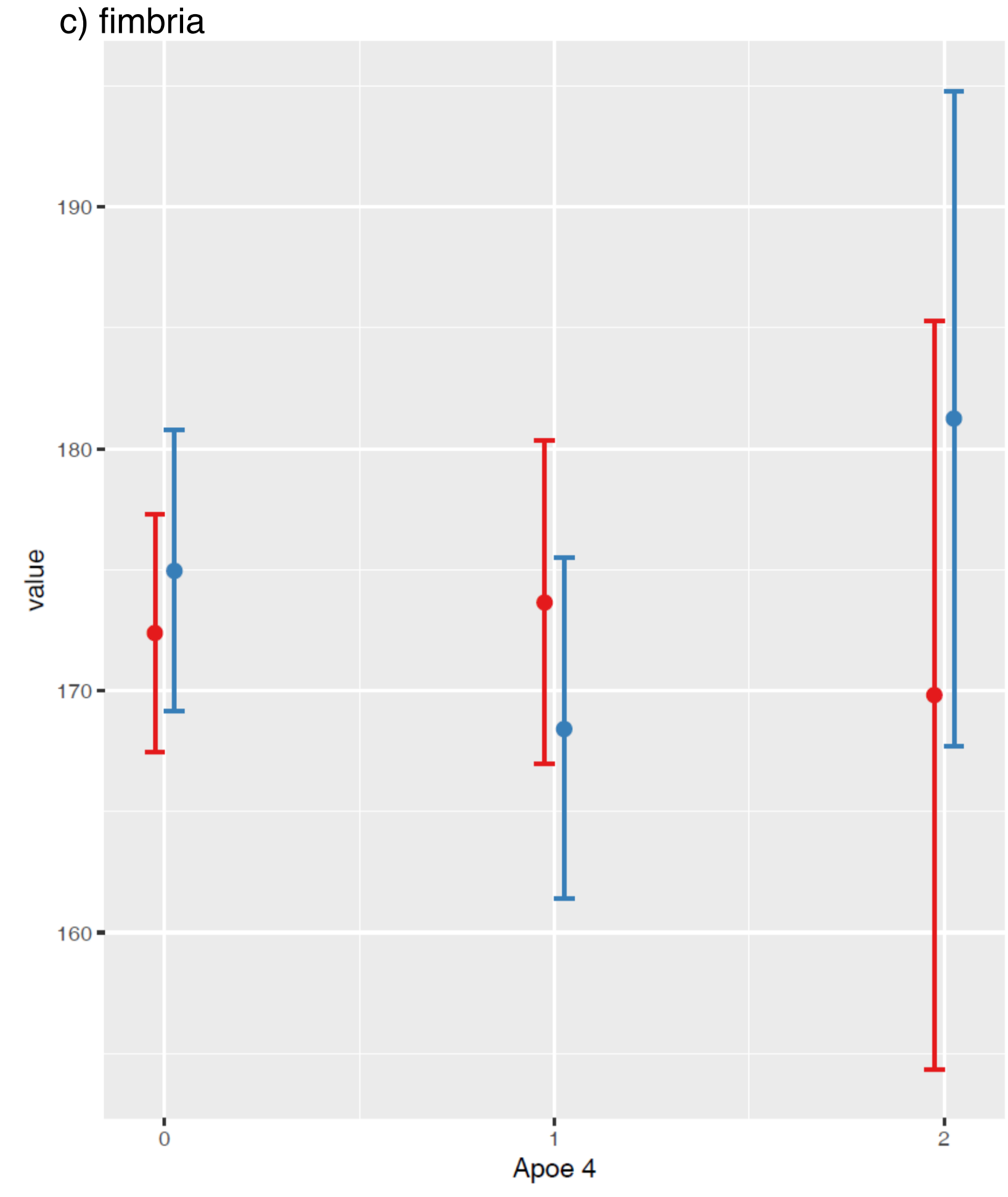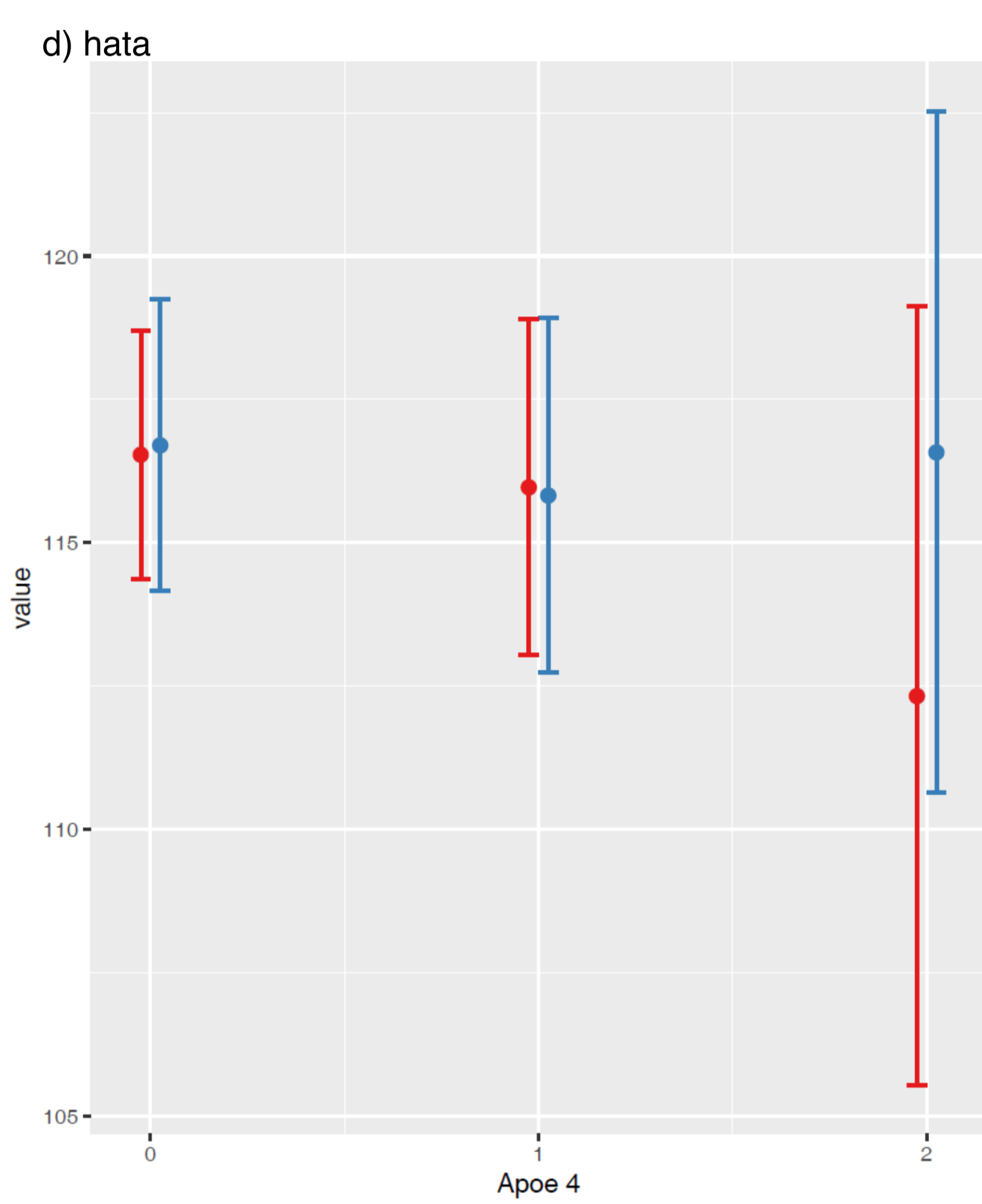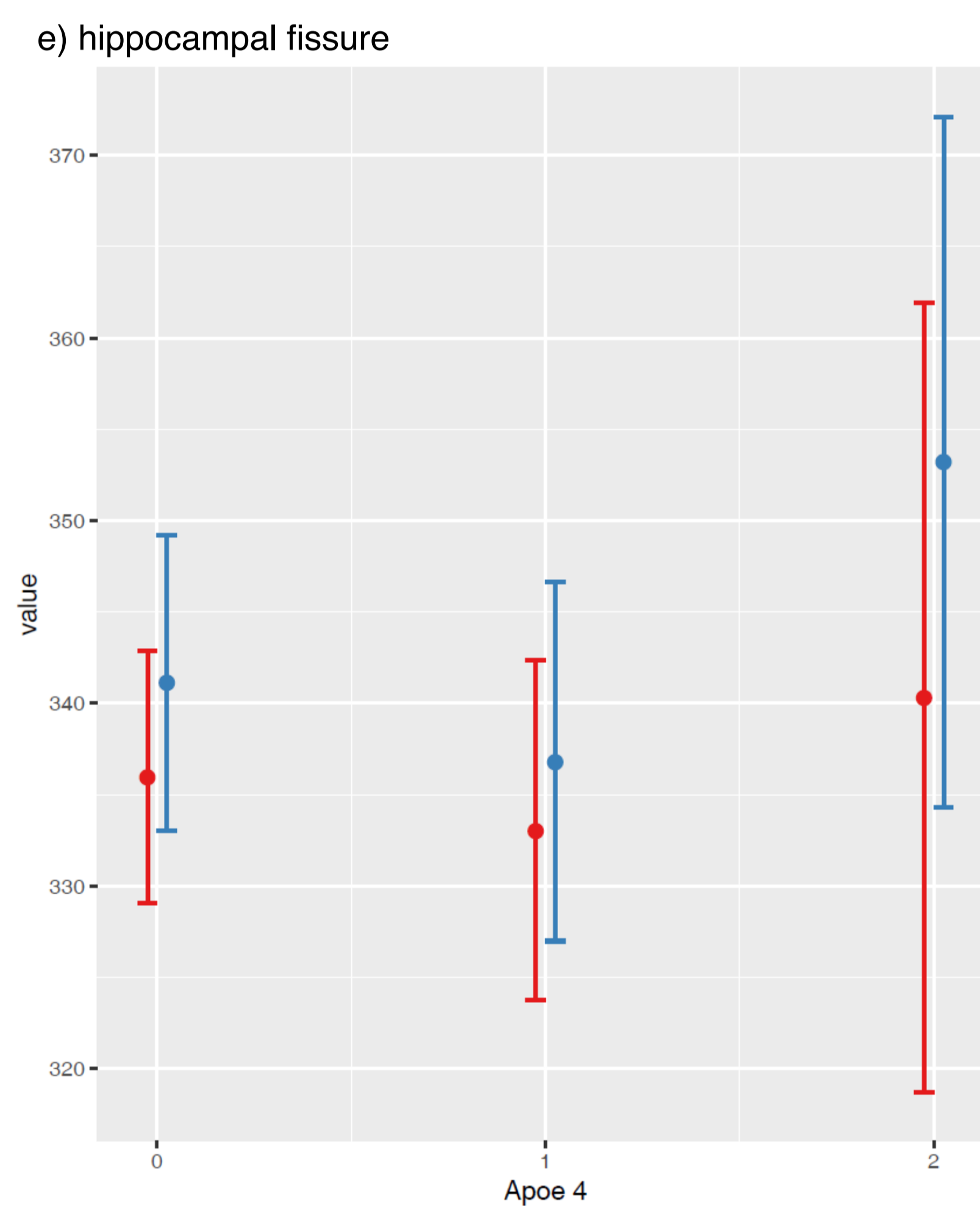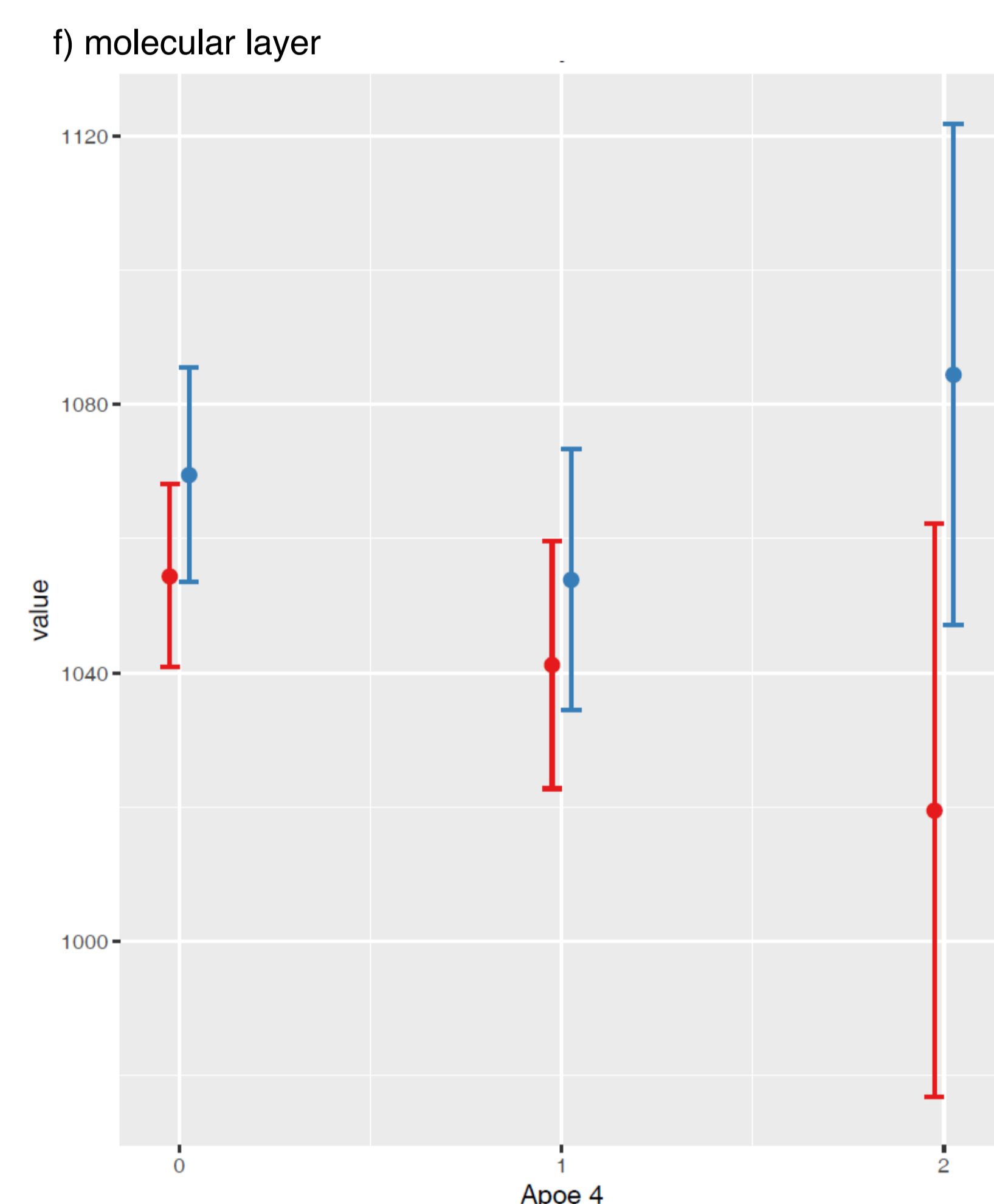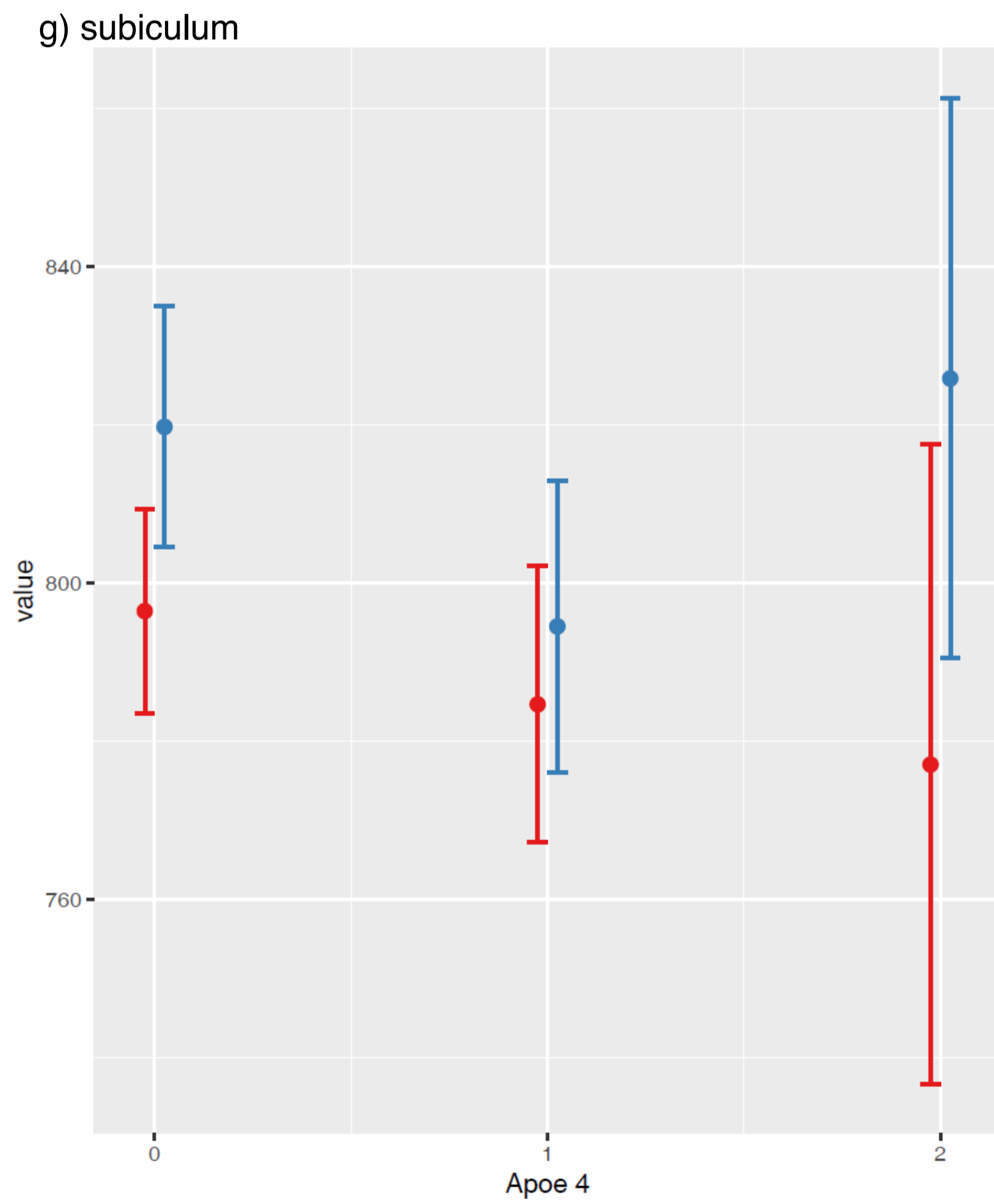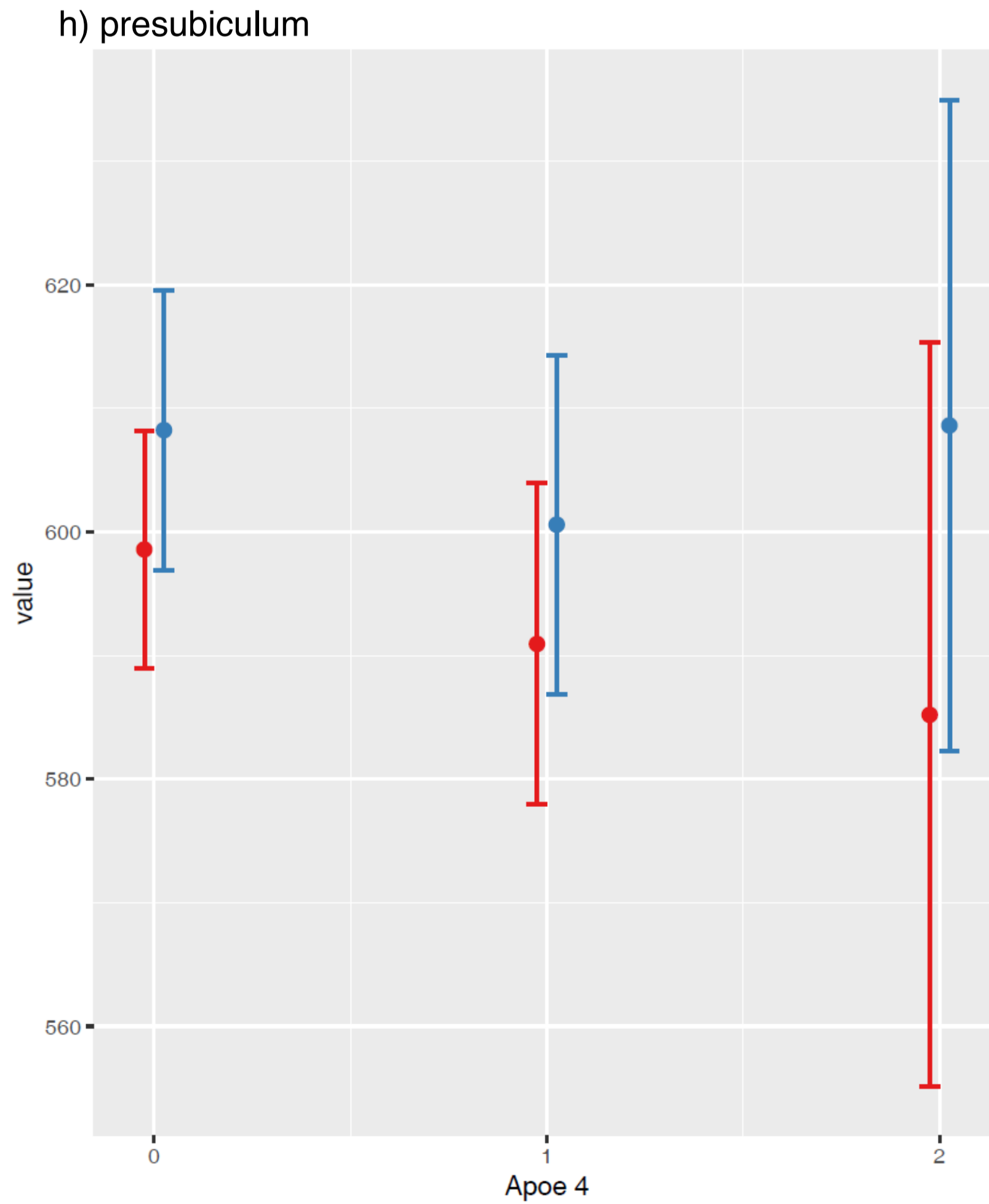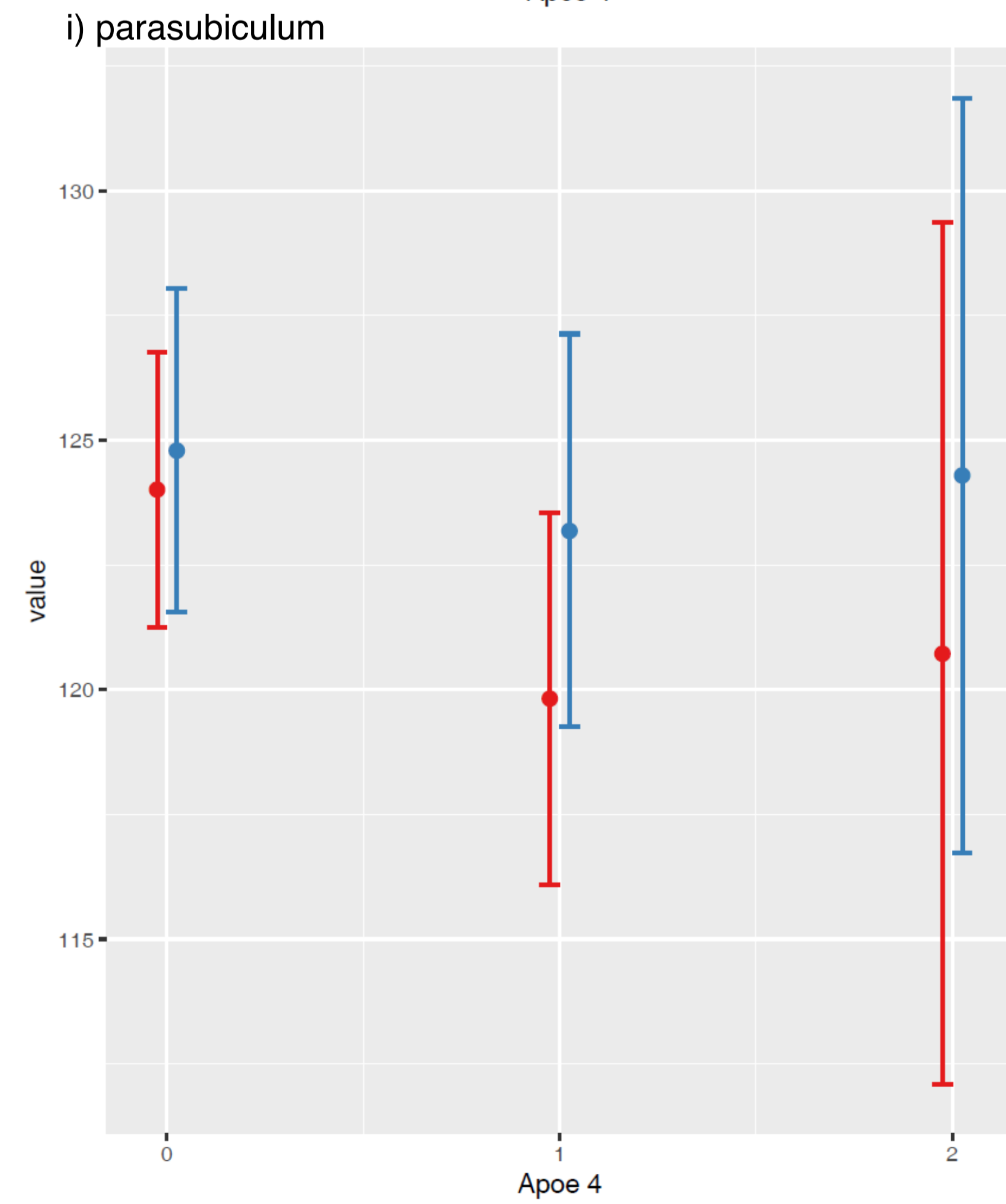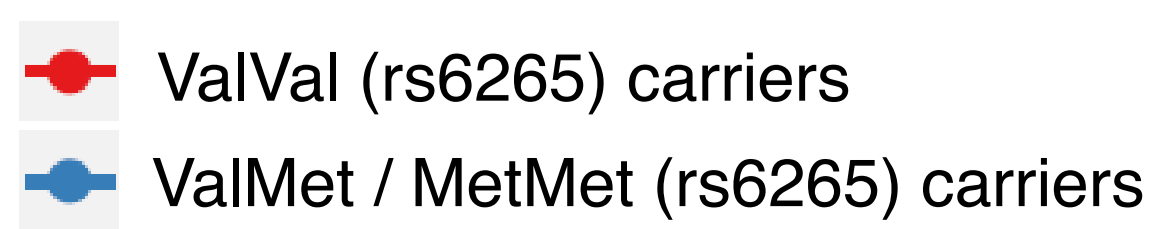

Supplement: Supplementary file 2 — Supplementary file2 (PDF 257 kb) Figure S2. Differences according BDNF Val66Met genotypes in associations between APOE-ε4 and the remaining subfields. Legend: CA1, cornu ammonis region 1; CA23, cornu ammonis region 23; hata, hippocampal-amygdaloid transition region. [file 429_2020_2125_MOESM2_ESM.pdf]
