# Supplementary material for: Effect of BDNF Val66Met on hippocampal subfields volumes and compensatory interaction with APOE-ε4 in middle-age cognitively unimpaired individuals from the ALFA study
Source: Brain Struct Funct. 2020 Aug 17;225(8):2331–45. doi: 10.1007/s00429-020-02125-3 (PMC7544723; doi:10.1007/s00429-020-02125-3)
Supplement: Supplementary file 3 — Supplementary file3 (DOCX 21 kb) Table S1. Main effects of Val66Met genotype on hippocampal subfields (mm3). All models were adjusted by sex, years of education, number of APOE-e4 allele and total intracranial volume. [file 429_2020_2125_MOESM3_ESM.docx]

|  | **Dominant genetic model** | | | | | |
| --- | --- | --- | --- | --- | --- | --- |
| **Hippocampal subfield** | **Effect (mm3)** | **CI 95%** | **Effect (%)** | **pvalue** | **FDR95%** | **AIC** |
| *CA1* | 18,575 | (-0.67, 37.82) | 1,54% | 0,059 | 0,531 | 5188,564 |
| *CA23* | 2,775 | (-4.26, 9.81) | 0,76% | 0,44 | 1 | 4323,665 |
| *CA4* | 5,255 | (-1.33, 11.84) | 1,15% | 0,118 | 0,944 | 4265,967 |
| *GC-ML-DG* | 5,515 | (-2.07, 13.1) | 1,02% | 0,155 | 1 | 4387,611 |
| *subiculum* | 20,527 | (6.87, 34.18) | 2,53% | **0,003** | **0.039*** | 4893,206 |
| *presubiculum* | 10,556 | (0.44, 20.67) | 1,74% | **0,041** | 0,41 | 4635,326 |
| *parasubiculum* | 1,845 | (-1.06, 4.75) | 1,46% | 0,214 | 1 | 3562,288 |
| *hippocampal fissure* | 5,226 | (-2.05, 12.5) | 1,52% | 0,16 | 1 | 4351,502 |
| *hippoccampal tail* | 12,37 | (-9.43, 34.17) | 1,15% | 0,267 | 1 | 5295,52 |
| *fimbria* | 0,568 | (-4.65, 5.79) | 0,32% | 0,831 | 1 | 4066,024 |
| *hata* | 0,333 | (-1.95, 2.61) | 0,28% | 0,775 | 1 | 3354,183 |
| *molecular layer* | 17,585 | (3.2, 31.97) | 1,64% | **0,017** | 0,204 | 4938,048 |
| *whole hippocampus* | 95,904 | (12.06, 179.75) | 1,46% | **0,025** | 0,275 | 6454,188 |
|  | **Additive genetic model** | | | | | |
| **Hippocampal subfield** | **Effect (mm3)** | **CI 95%** | **Effect (%)** | **pvalue** | **FDR95%** | **AIC** |
| *CA1* | 16,838 | (0.77, 32.9) | 1,40% | **0,041** | 0,41 | 5189,81 |
| *CA23* | 3,923 | (-1.95, 9.8) | 1,08% | 0,191 | 0,955 | 4324,523 |
| *CA4* | 5,618 | (0.13, 11.11) | 1,23% | **0,045** | 0,41 | 4265,973 |
| *GC-ML-DG* | 6,177 | (-0.14, 12.5) | 1,15% | 0,056 | 0,41 | 4387,38 |
| *subiculum* | 19,435 | (8.06, 30.81) | 2,39% | **0,001** | **0.013*** | 4890,434 |
| *presubiculum* | 8,491 | (0.04, 16.94) | 1,40% | **0,05** | 0,41 | 4635,584 |
| *parasubiculum* | 1,546 | (-0.88, 3.97) | 1,23% | 0,213 | 0,955 | 3562,742 |
| *hippocampal fissure* | 5,019 | (-1.05, 11.09) | 1,46% | 0,106 | 0,636 | 4352,85 |
| *hippoccampal tail* | 6,305 | (-11.92, 24.53) | 0,59% | 0,498 | 1 | 5298,305 |
| *fimbria* | 0,619 | (-3.74, 4.98) | 0,35% | 0,781 | 1 | 4066,089 |
| *hata* | 0,19 | (-1.72, 2.09) | 0,16% | 0,845 | 1 | 3355,804 |
| *molecular layer* | 16,578 | (4.59, 28.57) | 1,55% | **0,007** | 0,084 | 4937,902 |
| *whole hippocampus* | 85,72 | (15.75, 155.69) | 1,31% | **0,017** | 0,187 | 6454,65 |
|  | **Recessive genetic model** | | | | | |
| **Hippocampal subfield** | **Effect (mm3)** | **CI 95%** | **Effect (%)** | **pvalue** | **FDR95%** | **AIC** |
| *CA1* | 27,893 | (-15.32, 71.11) | 2,32% | 0,207 | 1 | 5190,558 |
| *CA23* | 14,291 | (-1.43, 30.01) | 3,93% | 0,076 | 0,684 | 4321,049 |
| *CA4* | 14,04 | (-0.69, 28.77) | 3,07% | 0,062 | 0,671 | 4264,9 |
| *GC-ML-DG* | 16,756 | (-0.19, 33.71) | 3,11% | 0,053 | 0,636 | 4385,858 |
| *subiculum* | 36,773 | (6.08, 67.47) | 4,53% | **0,019** | 0,247 | 4896,368 |
| *presubiculum* | 8,103 | (-14.65, 30.86) | 1,33% | 0,486 | 1 | 4639,059 |
| *parasubiculum* | 1,856 | (-4.66, 8.37) | 1,47% | 0,577 | 1 | 3563,544 |
| *hippocampal fissure* | 9,875 | (-6.43, 26.17) | 2,88% | 0,236 | 1 | 4352,082 |
| *hippoccampal tail* | -16,721 | (-65.59, 32.15) | -1,55% | 0,503 | 1 | 5296,322 |
| *fimbria* | 1,601 | (-10.09, 13.29) | 0,91% | 0,789 | 1 | 4065,996 |
| *hata* | -0,307 | (-5.42, 4.8) | -0,26% | 0,906 | 1 | 3354,253 |
| *molecular layer* | 30,991 | (-1.31, 63.29) | 2,90% | 0,061 | 0,671 | 4940,255 |
| *whole hippocampus* | 135,276 | (-53.2, 323.75) | 2,07% | 0,16 | 1 | 6457,253 |

|  | **Codominant genetic model** | | | | | | | | | | |
| --- | --- | --- | --- | --- | --- | --- | --- | --- | --- | --- | --- |
| **Hippocampal subfield** | **Effect (ValMet vs ValVal) (mm3)** | **CI 95% (ValMet vs ValVal)** | **Effect (ValMet vs ValVal) (%)** | **pvalue (ValMet vs ValVal)** | **FDR95% (ValMet vs ValVal)** | **Effect (MetMet vs ValVal) (mm3)** | **Effect (MetMet vs ValVal) (%)** | **CI 95% (MetMet vs ValVal)** | **pvalue (MetMet vs ValVal)** | **FDR95% (MetMet vs ValVal)** | **AIC** |
| *CA1* | 16,447 | (-3.51, 36.4) | 1,37% | 0,107 | 0,963 | 34,725 | 2,88% | (-9.19, 78.64) | 0,122 | 0,854 | 5189,91 |
| *CA23* | 1,187 | (-6.09, 8.47) | 0,33% | 0,749 | 1 | 14,802 | 4,07% | (-1.22, 30.82) | 0,071 | 0,639 | 4322,945 |
| *CA4* | 3,881 | (-2.93, 10.69) | 0,85% | 0,265 | 1 | 15,67 | 3,43% | (0.68, 30.66) | **0,041** | 0,418 | 4265,634 |
| *GC-ML-DG* | 3,82 | (-4.02, 11.66) | 0,71% | 0,34 | 1 | 18,368 | 3,41% | (1.11, 35.63) | **0,038** | 0,418 | 4386,933 |
| *subiculum* | 17,422 | (3.3, 31.54) | 2,15% | **0,016** | 0,208 | 43,99 | 5,42% | (12.92, 75.06) | **0,006** | 0,078 | 4892,451 |
| *presubiculum* | 10,317 | (-0.18, 20.81) | 1,70% | 0,055 | 0,588 | 12,39 | 2,04% | (-10.7, 35.48) | 0,294 | 1 | 4637,296 |
| *parasubiculum* | 1,748 | (-1.27, 4.76) | 1,39% | 0,256 | 1 | 2,579 | 2,05% | (-4.05, 9.21) | 0,446 | 1 | 3564,229 |
| *hippocampal fissure* | 4,372 | (-3.17, 11.91) | 1,27% | 0,256 | 1 | 11,69 | 3,41% | (-4.9, 28.28) | 0,168 | 1 | 4352,768 |
| *hippoccampal tail* | 15,386 | (-7.2, 37.97) | 1,43% | 0,183 | 1 | -10,318 | -0,96% | (-60.02, 39.39) | 0,684 | 1 | 5296,512 |
| *fimbria* | 0,41 | (-5, 5.82) | 0,23% | 0,882 | 1 | 1,784 | 1,02% | (-10.13, 13.7) | 0,769 | 1 | 4067,974 |
| *hata* | 0,397 | (-1.97, 2.76) | 0,34% | 0,742 | 1 | -0,136 | -0,12% | (-5.34, 5.07) | 0,959 | 1 | 3356,144 |
| *molecular layer* | 14,992 | (0.1, 29.88) | 1,40% | **0,049** | 0,588 | 37,231 | 3,48% | (4.46, 70) | **0,027** | 0,324 | 4938,311 |
| *whole hippocampus* | 86,007 | (-0.91, 172.93) | 1,31% | 0,053 | 0,588 | 171,084 | 2,61% | (-20.19, 362.36) | 0,08 | 0,64 | 6455,441 |

*Legend: CA1, cornu ammonis region 1; CA23, cornu ammonis region 23; CA4, cornu ammonis region 4; GC-ML-DG, granule cells in the molecular layer of the dentate gyrus; hata, hippocampal-amygdaloid transition region; HP, hippocampus; CI95, confidence interval; FDR95, False Discovery Rate corrected P-value < 0.05.*

**Table S1**. Main effects of Val66Met genotype on hippocampal subfields (mm3). All models were adjusted by sex, years of education, number of APOE-e4 allele and total intracranial volume.
