# Supplementary material for: Effect of BDNF Val66Met on hippocampal subfields volumes and compensatory interaction with APOE-ε4 in middle-age cognitively unimpaired individuals from the ALFA study
Source: Brain Struct Funct. 2020 Aug 17;225(8):2331–45. doi: 10.1007/s00429-020-02125-3 (PMC7544723; doi:10.1007/s00429-020-02125-3)
Supplement: Supplementary file 4 — Supplementary file4 (DOCX 14 kb) Table S2. Main effects of number of APOE-e4 alleles on hippocampal subfields (mm3). All models were adjusted by sex, years of education, age, val66Met genotypes and total intracranial volume. [file 429_2020_2125_MOESM4_ESM.docx]

**Table S2.** Main effects of number of APOE-e4 alleles on hippocampal subfields (mm3). All models were adjusted by sex, years of education, age, val66Met genotypes and total intracranial volume.

| **Hippocampal subfield** | **Effect (mm3)** | **CI 95%** | **Effect (%)** | **pvalue** | **FDR95%** |  |
| --- | --- | --- | --- | --- | --- | --- |
|  |  |  |  |  |  |  |
| *CA1* | -10.058 | (-25.61, 5.5) | -0.84% | 0.206 | 1 |  |
|  |  |  |  |  |  |  |
| *CA23* | -1.268 | (-6.95, 4.42) | -0.35% | 0.662 | 1 |  |
|  |  |  |  |  |  |  |
| *CA4* | -2.508 | (-7.82, 2.81) | -0.55% | 0.355 | 1 |  |
|  |  |  |  |  |  |  |
| *GC-ML-DG* | -2.946 | (-9.07, 3.17) | -0.55% | 0.346 | 1 |  |
|  |  |  |  |  |  |  |
| *subiculum* | -9.866 | (-20.88, 1.14) | -1.22% | 0.08 | 1 |  |
|  |  |  |  |  |  |  |
| *presubiculum* | -5.125 | (-13.31, 3.06) | -0.84% | 0.22 | 1 |  |
|  |  |  |  |  |  |  |
| *parasubiculum* | -1.941 | (-4.29, 0.41) | -1.54% | 0.106 | 1 |  |
|  |  |  |  |  |  |  |
| *hippocampal fissure* | 0.54 | (-5.34, 6.42) | 0.16% | 0.857 | 1 |  |
|  |  |  |  |  |  |  |
| *hippoccampal tail* | -15.298 | (-32.94, 2.35) | -1.42% | 0.09 | 1 |  |
|  |  |  |  |  |  |  |
| *fimbria* | -0.347 | (-4.57, 3.87) | -0.20% | 0.872 | 1 |  |
|  |  |  |  |  |  |  |
| *hata* | -0.838 | (-2.68, 1.01) | -0.71% | 0.374 | 1 |  |
|  |  |  |  |  |  |  |
| *molecular layer* | -8.994 | (-20.6, 2.62) | -0.84% | 0.13 | 1 |  |
|  |  |  |  |  |  |  |
| *whole hippocampus* | -59.19 | (-126.94, 8.56) | -0.90% | 0.088 | 1 |  |
| *Legend: CA1, cornu ammonis region 1; CA23, cornu ammonis region 23; CA4, cornu ammonis region 4; GC-ML-DG, granule cells in the molecular layer of the dentate gyrus; hata, hippocampal-amygdaloid transition region; HP, hippocampus; CI95, confidence interval; FDR95, FDR corrected P-value < 0.05.* | | | | | | |
